# Cerebellar Neurodegeneration and Neuronal Circuit Remodeling in Golgi pH Regulator-Deficient Mice

**DOI:** 10.1523/ENEURO.0427-18.2019

**Published:** 2019-05-29

**Authors:** Yu-shin Sou, Soichiro Kakuta, Yuji Kamikubo, Kazue Niisato, Takashi Sakurai, Laxmi Kumar Parajuli, Isei Tanida, Hiromitsu Saito, Noboru Suzuki, Kenji Sakimura, Yusuke Maeda, Taroh Kinoshita, Yasuo Uchiyama, Masato Koike

**Affiliations:** 1 Department of Cell Biology and Neuroscience, Juntendo University Graduate School of Medicine, Bunkyo, Tokyo 113-8421, Japan; 2 Laboratory of Morphology and Image Analysis, Research Support Center, Juntendo University Graduate School of Medicine, Bunkyo, Tokyo 113-8421, Japan; 3 Department of Cellular and Molecular Neuropathology, Juntendo University Graduate School of Medicine, Bunkyo, Tokyo 113-8421, Japan; 4 Department of Pharmacology, Juntendo University Graduate School of Medicine, Bunkyo, Tokyo 113-8421, Japan; 5 Functional Genomics Institute, Life Science Research Center, Mie University, Tsu, Mie 514-8507, Japan; 6 Department of Cellular Neurobiology, Brain Research Institute, Niigata University, Niigata 951-8585, Japan; 7 Research Institute for Microbial Diseases, Osaka University, Suita, Osaka 565-0871, Japan; 8 Advanced Research Institute for Health Science, Juntendo University, Bunkyo, Tokyo 113-8421, Japan

**Keywords:** cerebellar circuit, Golgi apparatus, Golgi fragmentation, Golgi pH regulator, neurodegeneration, Purkinje cell

## Abstract

The Golgi apparatus plays an indispensable role in posttranslational modification and transport of proteins to their target destinations. Although it is well established that the Golgi apparatus requires an acidic luminal pH for optimal activity, morphological and functional abnormalities at the neuronal circuit level because of perturbations in Golgi pH are not fully understood. In addition, morphological alteration of the Golgi apparatus is associated with several neurodegenerative diseases, including Parkinson’s disease, Alzheimer’s disease, and amyotrophic lateral sclerosis. Here, we used anatomical and electrophysiological approaches to characterize morphological and functional abnormalities of neuronal circuits in Golgi pH regulator (GPHR) conditional knock-out mice. Purkinje cells (PCs) from the mutant mice exhibited vesiculation and fragmentation of the Golgi apparatus, followed by axonal degeneration and progressive cell loss. Morphological analysis provided evidence for the disruption of basket cell (BC) terminals around PC soma, and electrophysiological recordings showed selective loss of large amplitude responses, suggesting BC terminal disassembly. In addition, the innervation of mutant PCs was altered such that climbing fiber (CF) terminals abnormally synapsed on the somatic spines of mutant PCs in the mature cerebellum. The combined results describe an essential role for luminal acidification of the Golgi apparatus in maintaining proper neuronal morphology and neuronal circuitry.

## Significance Statement

The Golgi luminal acidic condition is essential for normal activity of the Golgi apparatus and its dysregulation contributes to several human diseases. In neurons, however, pharmacological tools cannot be used for *in vivo* studies because they lack specificity. Here, the *in vivo* role of Golgi luminal acidification by inactivation of the Golgi pH regulator (GPHR) in cerebellar neurons is reported. The GPHR is indispensable for neuronal survival, normal Golgi morphology and axonal integrity. Intriguingly, after formation of Pinceau structures, basket cells (BCs) exhibited significant loss of function, resulting in an alteration of neuronal circuitry around Purkinje cells (PCs). These results highlight an essential role of the Golgi luminal pH in the maintenance of gross axonal morphology and subcellular structures in PCs.

## Introduction

The flattened, cisternal structure of the Golgi apparatus is essential for ensuring that proteins are correctly glycosylated and sorted to the appropriate destination (Klumperman, 2011; Papanikou and Glick, 2014). The optimal activity of intracellular organelles, such as the Golgi apparatus, is strictly regulated by the acidic pH of the lumen (Weisz, 2003). Perturbation of the luminal pH not only has marked effects on posttranslational modification and transport of proteins but also causes morphological abnormalities in the Golgi apparatus (Palokangas et al., 1994; Kellokumpu et al., 2002). Similar morphologic abnormalities have also been observed in cerebellar Purkinje cells (PCs; Baloyannis, 2014) and motor neurons in patients with neurodegenerative disorders (Gonatas et al., 1992; Mourelatos et al., 1996; Haase and Rabouille, 2015; Ayala and Colanzi, 2017). However, the causative link between impaired luminal acidification of the Golgi apparatus, fragmentation of the Golgi apparatus and neurodegeneration remains unresolved.

Several hypotheses have been proposed to explain how fragmentation of the Golgi apparatus can cause neurodegeneration. Some of these include microtubule depolymerization, pathogenic protein misfolding, mislocalization of aggregate-prone proteins and phosphorylation of GRASP65, a structural protein of the Golgi apparatus (Alvarez-Miranda et al., 2015). In addition to these explanations, we hypothesized that the impairment of luminal acidification of the Golgi apparatus causes fragmentation, which results in neurodegeneration. Ubiquitin protein ligase E3A (Ube3a), a causative gene product of Angelman’s syndrome, is involved in the acidification of the Golgi lumen (Condon et al., 2013). Interestingly, the Golgi-associated protein, ATP6V0A2, is involved in congenital disorders of glycosylation (CDG) because of a pH imbalance in the Golgi apparatus (Kornak et al., 2008; Demaegd et al., 2013). Additionally, patients with several types of CDG as a result of mutations in genes encoding Golgi-associated proteins show cerebellar atrophy and ataxia (Climer et al., 2015).

We previously identified an anion channel that is localized to the Golgi apparatus and is a Golgi pH regulator (GPHR). The GPHR is essential for maintaining Golgi luminal acidification (Maeda et al., 2008). Using keratinocyte-specific *GPHR* knock-out mice, the GPHR was found to be essential for proper skin barrier function (Tarutani et al., 2012). Here, we took advantage of the GPHR conditional knock-out mice to better understand the morphologic and functional abnormalities in cerebellar circuits. We show that deletion of *GPHR* in cerebellar inhibitory circuits [PCs and basket cells (BCs)] leads to cerebellar ataxia due to PC degeneration. Impaired luminal acidification of the Golgi apparatus leads to vesiculation and fragmentation in PCs. Importantly, we found that axonal and synaptic abnormalities precede the loss of PCs. Moreover, the loss of *GPHR* in BCs causes disruption of inhibitory input to PC soma and further leads to climbing fiber (CF) innervation onto the soma of mutant PCs. These results indicate that GPHR-dependent Golgi luminal acidification plays an essential role in the structural and functional maintenance of PCs and BCs.

## Materials and Methods

### Animals

Male *GPHR^F/F^* mice (Tarutani et al., 2012) and female *GluD2-Cre* knock-in mice (Yamasaki et al., 2011) were crossed to produce *GPHR^F/F^;GluD2-Cre* mice. Male *GPHR^F/F^* mice and female L7/pcp2-*Cre* knock-in mice (Saito et al., 2005) were crossed to produce *GPHR^F/F^;L7-Cre* mice. In the present study, *GPHR^F/+^* mice expressing Cre recombinase were used as the control. The procedures involving animal care, surgery and sample preparation were approved by the Animal Experimental Committee of Juntendo University and performed in accordance with the guidelines for the Care and Use of Laboratory Animals. Mice were housed in specific pathogen-free conditions with 12/12 h light/dark cycles at Juntendo University.

### Antibodies

Preparation of rabbit anti-GPHR and anti-mGluR1α antibodies were described previously (Maeda et al., 2008; Kamikubo et al., 2013). Goat anti-calbindin [(Af1040), RRID: AB_2571569], guinea pig anti-calbindin [(Af-280), RRID: AB_2571570], anti-parvalbumin [(Af-1000), RRID: AB_2571615], rabbit anti-GABA_A_ receptor α1 subunit [GABA_A_ Rα1; (Af-660), RRID: AB_2571571], anti-vesicular GABA transporter [vGAT; (Af-500), RRID: AB_2571622], anti-type-1 vesicular glutamate transporter [vGlut1; (Af-280), RRID: AB_2571616], and anti-vGlut2 [(Af-720), RRID: AB_2571619] were purchased from the Frontier Institute. Mouse anti-NeuN [(A60), RRID: AB_2298772] was purchased from Millipore. Rat anti-Mac 2 [(M3/38), RRID: AB_10060357] was purchased from Cedarlane. Rabbit anti-IbaI [(019-19741), RRID: AB_839504] was purchased from Wako Pure Chemical Industries. Mouse anti-GM130 [(35/GM130), RRID: AB_398141] was purchased from BD Biosciences. Sheep anti-TGN38 [(AAHP499), RRID: AB_2287346] was purchased from AbD Serotec. A rat monoclonal antibody against GPHR was raised in rat using the peptide Cys-AHKQAPEKHMAP as the antigen. Rat hybridoma cells (Clone 35C5) were obtained by standard approaches.

### Plasmids

The plasmid containing the open reading frame of the pH sensor protein, pHluorin, was kindly provided by Dr. James Edward Rothman (Miesenböck et al., 1998). We used the N-terminal 81 amino acids of human beta 1,4-galactosyltransferase as a targeting signal for the Golgi apparatus. The plasmids, pHluorin-Golgi and pmKate2-Golgi, were constructed based on pEYFP-Golgi (TAKARA/Clontech, catalog #6909-1) by replacing the sequence encoding EYFP with the sequences encoding pHluorin and mKate2, respectively (Tanida et al., 2014). The DNA fragment encoding pHluorin-Golgi was introduced into the NheI-EcoRI sites of pIRES, and the DNA fragment encoding mKate2-Golgi was introduced into the SalI-NotI sites of pIRES (TAKARA/Clontech, catalog #Z1605N). The resultant plasmid was named as pHluorin-Golgi-IRES-mKate2-Golgi.

### Primary culture of cortical neurons and adenovirus infection

Primary cortical neurons were prepared according to a general protocol. Briefly, the cortex was dissected and dissociated from E14 mice embryos. Dissociated cortical cells were plated on poly-L-Ornithine (Sigma-Aldrich) coated 14-mm coverslips. Neurobasal medium (Thermo Fisher Scientific) supplemented with 2% B27 (Thermo Fisher Scientific) and 2 mmol L-glutamine (Nacalai Tesque) was used as the culture medium. After 2 d, 10 mmol/L cytosine arabinoside (Nacalai Tesque) was added to inhibit non-neuronal growth. Two days after this step, with the aim of achieving equal infection, the culture medium containing the adenovirus with 10 multiplicity of infection (MOI) was added to the medium. The lysates from cultured neurons were subjected to immunoblotting to detect GPHR and actin. Adenovirus vectors: Ad-CMV-iCre (#1045) and Ad-GFP (#1060) were purchased from Vector Biolabs.

### Quantitative real-time PCR

cDNA was synthesized from 1 µg of total RNA using the Transcriptor First-Strand cDNA synthesis kit (Roche Applied Science). Quantitative PCR was performed using the SYBR Green Real-time PCR Master Mix (Toyobo) with a Thermal Cycler Dice Real Time System II (TAKARA BIO). The signal intensity was normalized to the reference β-actin signal. The following primer sequences were used: *GPHR* left, 5′-CATGGTGCCCTTCTACATTG-3′; *GPHR* right, 5′-TCCCAGCTTCCAGAAGAAGT-3′; *β-actin* left, 5′-GCAAGCAGGAGTACGATGAG-3′; *β-actin* right, 5′-TCCCAGCTTCCAGAAGAAGT-3′.

### Measurement of pHluorin intensity

pHluorin-Golgi and mKate2-Golgi were expressed by adenovirus-mediated transfection of pHluorin-Golgi-IRES-mKate2-Golgi in primary cultured neurons using Lipofectamine 2000 (Thermo Fisher Scientific). Seven days after transfection, live-cell images of primary cultured neurons expressing pHluroin-Golgi-IRES-mKate2-Golgi were obtained using a FV1000 confocal laser scanning microscope (Olympus). The intensities of pHluorin and mKate2 in the same ROI were measured by the histogram analysis plugin in ImageJ (Schneider et al., 2012). The mean of the intensity ratio (pHluorin/mKate2) was calculated and processed for statistical analysis.

### Histological examination

Deeply anesthetized mice were fixed by cardiac perfusion with 4% paraformaldehyde in 0.1 M phosphate buffer (pH 7.4). Tissues were embedded in paraffin or OCT-compound (Tissue-Tek, 4583; Sakura Fintek). Three-micron paraffin sections were stained with 0.1% cresyl violet for Nissl staining. Meyer’s hematoxylin and eosin (H & E) staining was performed on 20-µm-thick cryosections. For immunofluorescence, frozen sections were subjected to antigen retrieval by incubating in target retrieval solution (S1699, Dako) for 10 min at 105 °C and blocked with TNB buffer (0.1 M Tris-HCl, pH 7.5, 0.15 M NaCl, and 0.5% TSA blocking reagent; FP102; PerkinElmer) at room temperature for 1 h. The sections were then incubated with a mixture of primary antibodies at 4 °C overnight, followed by incubation with fluorescently labeled secondary antibodies [Alexa Fluor 488 (Thermo Fisher Scientific) for Cy3 or Cy5 (Jackson ImmunoResearch)] containing DAPI at room temperature for 1 h. Fluorescence images were obtained using a FV1000 confocal laser scanning microscope (Olympus).

### Stereological sectioning and quantification of PCs

The total number of PCs in the mouse cerebellum was estimated by stereological quantification and optical disector counting methods, as described previously (Chintawar et al., 2009, 2012; Ip et al., 2017). Sagittal cerebellar cryosections from control and *GPHR^F/F^;GluD2-Cre* mice (three mice per group) were serially cut at 20-µm thicknesses to cover the entire cerebellum. Systematic random sampling of every 20th section collected was performed for a total of 18 sections per mice. Sections were immunostained with the anti-calbindin antibody to reveal PC soma and their dendrite in the molecular layer. Calbindin immunostaining images were visualized by using a fluorescence microscope BZ-X710 with a 20× objective lens (Keyence). The optical disector method was applied to identify and count PC tops and thus to calculate average PC densities (*Nv*). The reference volume (*Vref*) from PCs and molecular layers was obtained using Cavalieri’s principle. Finally, the total number of PCs (*Npc*) was estimated according to the formula: *Npc = Vref × Nv*. The images were analyzed using ImageJ cell counter and Grid layout plugins (Schneider et al., 2012).

### Electron microscopy (EM)

Mice were fixed by cardiac perfusion with 4% paraformaldehyde in 0.1 M phosphate buffer (pH 7.4). Cerebellar sections were post fixed with 2% paraformaldehyde and 2% glutaraldehyde in 0.1 M phosphate buffer (pH 7.4) overnight followed by post fixation with 1% OsO_4_, dehydration with a graded series of ethanol and embedding in Epon812 (Oken Shoji). Ultrathin sections were cut with an ultramicrotome UC6 (Leica Microsystems), stained with uranyl acetate and lead citrate and examined with a transmission EM HT7700 (Hitachi). For volume EM, images were taken with a focused ion beam scanning EM (FIB-SEM) Helios Nanolab 660 (FEI Company). Epon blocks were milled by a focused ion beam and a serial block-face for every 20 nm was imaged using a backscattered electron detector (MD detector) at an acceleration voltage of 2.0 kV and a current of 0.4 nA. 3D reconstruction was performed using Amira software (FEI Company).

### Measurement of the cerebellar area, PC density, and microglia density

Measurement of the cerebellar area was conducted as described previously (Xu et al., 2018). Sagittal cerebellar cryosections (20 µm thick) from control and *GPHR^F/F^;GluD2-Cre* mice (three mice per group) were serially cut and every 10th section was collected for H & E staining. Cerebellar size was measured from five sections corresponding to positions 102–104 of the mouse brain atlas (Franklin and Paxinos, 2007). Sections were visualized by using a fluorescence microscope BZ-X710 with a 4× objective lens. Images were stitched into a whole cerebellum by the BZ-X image analyzer (Keyence) and the area of the cerebellum was measured. The mean area was calculated and processed for statistical analysis. For quantification of PC densities, cerebellar sections from control and *GPHR^F/F^;GluD2-Cre* mice (three mice per group) were immunostained with the anti-calbindin antibody. The number of calbindin-positive PCs from each cerebellum was counted from a total of five sections, corresponding to positions 102–104 of the mouse brain atlas (Franklin and Paxinos, 2007). The PC density was calculated as the total number of PC somata divided by the sum of the area of PC and molecular layers. The mean PC densities were calculated and processed for statistical analysis. To measure microglia density, sections containing cerebellar lobule IX from control and *GPHR^F/F^;GluD2-Cre* mice (three mice per group) were immunostained with the anti-IBA1 antibody, and nuclei were stained with DAPI. The number of total IBA1-positive cells was counted on five sections. The microglia density was obtained by dividing the IBA1-positive cells, with clearly visible nuclei, by the total area of the lobule. The mean density was obtained from all the microglia analyzed and processed for statistical analysis. All images were analyzed using ImageJ.

### Morphometry of PCs

To quantify immunofluorescence images, five sections containing cerebellar lobule IX from each mouse were immunostained and analyzed using ImageJ. Measurements were obtained from three mice in each group. To calculate the ratio of PCs in which the Golgi apparatus is abnormally distributed, the number of PCs with such abnormality was divided by the total number of PCs. The abnormal distribution of the Golgi apparatus in PCs was defined by perinuclear accumulation of GM130 and TGN38, as revealed by immunostaining. The mean ratio of PCs with abnormal distribution of the Golgi apparatus was calculated and processed for statistical analysis. Calbindin immunostaining images were analyzed by ImageJ to quantify axon swelling, which enabled measurement of the size and number of axon swellings in the immunostained sections. An axonal torpedo area exceeding 15 µm^2^ was defined as swelling axons. The number of swelling axons was divided by the area of granule cell layer or deep cerebellar nuclei (DCNs) to calculate the density of axonal swelling. To count Pinceau structures, sections were immunostained with anti-calbindin and anti-parvalbumin antibodies to elucidate BC axon terminals. The ratio of PCs with Pinceau structures was calculated as the total number of PCs with Pinceau structures divided by the total number of PCs. The ratio obtained was processed for statistical analysis. To quantify the frequency of mGluR1α and vGlut2 single-positive or double-positive puncta, analysis was restricted to the bottom two-thirds of the PC soma to avoid contamination of the analysis by puncta on dendrites. The ratio of vGlut2 puncta on PC soma was calculated by dividing the positive cells with the total number of cells analyzed. The mean obtained was processed for statistical analysis. For quantification of lamellar bodies, defined as smooth ERs with more than three stacks, in the cytoplasm of PCs, electron micrographs taken from control (*n* = 5, P60) and *GPHR^F/F^;GluD2-Cre* (*n* = 5, P60) mice were analyzed. The number of lamellar bodies was counted and divided by the total area of the PC cytoplasm to show lamellar body densities. The mean of lamellar body densities was calculated and processed for statistical analysis.

### Electrophysiology

Electrophysiological analysis was performed by a conventional approach (Nakayama et al., 2012). Briefly, mice were decapitated following CO_2_ anesthesia, and the brains were rapidly removed and placed in chilled modified external solution (0 °C−4 °C) containing: 120 mM choline-Cl, 2 mM KCl, 8 mM MgCl_2_, 28 mM NaHCO_3_, 1.25 mM NaH_2_PO_4_, and 20 mM glucose, with bubbling of 95% O_2_ and 5% CO_2_, pH 7.4. Parasagittal cerebellar slices (250 μm in thickness) were prepared by using a vibratome slicer (VT-1200S, Leica). For recovery, slices were incubated for at least 1 h in normal artificial CSF (ACSF) composed of 125 mM NaCl, 2.5 mM KCl, 2 mM CaCl_2_, 1 mM MgSO_4_, 1.25 mM NaH_2_PO_4_, 26 mM NaHCO_3_, and 20 mM glucose, pH 7.4, which was bubbled continuously with a mixture of 95% O_2_ and 5% CO_2_ at room temperature. Whole-cell recordings were made from visually identified PCs using an upright microscope (BX51WI, Olympus) at 32 °C. The whole-cell pipette solution contained 140 mM CsCl, 10 mM 2-[4-(2-hydroxyethyl)-1-piperazinyl]ethanesulfonic acid (HEPES), 1 mM ethylene glycol-bis(2-aminoethylether)-N, N, N', N'-tetraacetic acid (EGTA), 4.6 mM MgCl_2_, 0.1 mM CaCl_2_, 4 mM Na-ATP, and 0.4 mM Na-GTP, pH 7.3, adjusted with 50% CsOH, 287 mOsm. Resistance of the patch pipette was 3–5 MΩ. When GABA_A_ receptor-mediated miniature IPSCs (mIPSCs) were recorded, tetrodotoxin (TTX; 1 μM) and 16-cyano-7-nitro-quinoxaline-2,3-dione (CNQX; 10 μM) were present to block action potentials and AMPA receptor-mediated currents. An Axopatch 1D amplifier (Molecular Devices) was used and the signal was filtered at 5 kHz, digitized at 10 kHz and stored on a personal computer (pClamp 10, Molecular Devices).

### Behavioral tests

The accelerating rotarod test was performed on a rotarod machine with automatic timers and falling sensors (MK-660D, Muromachi Kikai). Control and *GPHR^F/F^;GluD2-Cre* mice (*n* = 5 for each genotype of females) were placed on a rotating rod, rotation was accelerated to 40 rpm for 300 s, and the latency period for each mouse to fall off was measured. If a mouse remained on the rod for >300 s, the latency period was recorded as 300 s. Mice underwent three trials per day and the mean latency period of the three trials was considered for statistical analysis. Cerebellar ataxic phenotypes of control and *GPHR^F/F^;GluD2-Cre* mice (three mice per group) were evaluated at P45, P60, P90, P150, and P300 using the composite phenotype scoring system based on hindlimb clasping, open field gait and ledge tests (Guyenet et al., 2010). Each test was repeated three times. All tests were scored on a scale of 0–3, with a combined total of 0–9 for all three tests. For the limb clasping test, mice were lifted for 20 s by grasping their tail and movement of the hind limbs was scored as follows. A score of 0 indicates a mouse spreading their hind limbs away from the abdomen. A score of 1 indicates a mouse pulling their hindlimb partially toward their abdomen for >5 s. A score of 2 indicates a mouse pulling both hind limbs partially toward their abdomen for >5 s. A score of 3 indicates a mouse retracting hind limbs and touching the abdomen for >50% of the observation time. For the open field gait test, mice were placed on a flat surface and oriented away from the researcher. A score was recorded as follows. A score of 0 indicates that a mouse moved normally and its abdomen did not touch the surface. A score of 1 indicates that tremor was observed or the mouse appeared to limp while walking. A score of 2 indicates a mouse showing severe tremor, severe limp, lowered pelvis or feet pointing away from the body. A score of 3 indicates that a mouse had difficulty to move forward and dragged their abdomen along the surface. For the ledge test, mice were lifted from a cage and placed on the ledge of the cage. A score was recorded as follows. A score of 0 indicates that a mouse walked along the ledge without losing its balance and went back into the cage using its paws. A score of 1 indicates that a mouse lost its footing while walking on the ledge. A score of 2 indicates that a mouse was unable to walk effectively on or let itself down from the ledge. A score of 3 indicates that a mouse was unable to walk, get down from the ledge, or simply fell off. All behavioral tests were performed by researchers blinded to the genotype of the mice.

### Statistical analysis

See Table 1 for a summary of statistical results relating to this work. Actual *p* values and the number of samples are stated in the text, figure legends, and Table 1. All statistical analyses were performed using GraphPad Prism 6 (GraphPad Software Inc.; RRID: SCR_002798). At least three independent samples were used for assessing statistical significance. Normality of the datasets was determined by the Kolmogorov–Smirnov test. Comparisons between multiple experimental groups for the accelerating Rotarod test, measurement of cerebellar area, quantitative analysis of PC density, Pinceau structure counting and quantification of the frequency of vGlut2 were made using one-way ANOVA followed by Sidak’s multiple comparisons test. For comparing two experimental groups for quantitative real-time PCR, quantitative analysis of the pHluorin intensity, density of microglia, abnormal distribution of the Golgi apparatus, quantitative analysis of axonal swelling, electrophysiological analysis and quantitative analysis of the lamellar body density were conducted using the unpaired Student’s *t* test. For combined scores of the composite phenotype scoring system, which did not follow a normal distribution according to the Kolmogorov–Smirnov test, a non-parametric test was performed using the Kruskal–Wallis rank sum test followed by the Dunn’s test. The data are presented as the mean ± SEM; *p* < 0.05 was considered statistically significant and *p* < 0.01 was considered highly significant.

**Table1.** Summary of statistical analyses

Figure, statistical test, and measure								
Fig. 1*B*								
Quantitative real-time PCR		*GPHR^F/F^*AV: GFP			*GPHR^F/F^*AV: Cre			
unpaired Student’s *t* test		mRNA levels of *GPHR*			mRNA levels of *GPHR*			
		Mean	SEM	*n*	Mean	SEM	*n*	Statistics
Relative mRNA levels		0.992	± 0.025	6	0.044	± 0.011	6	*p* < 0.0001
Fig. 1*D*								
Intensity of pHluorin		*GPHR^F/+^*AV: Cre			*GPHR^F/F^*AV: Cre			
unpaired Student’s *t* test		Mean	SEM	*n*	Mean	SEM	*n*	
Raito (pHluorin/mKate2; 488/562 nm)		1.015	± 0.038	5	1.390	± 0.068	5	*p* = 0.0013
Fig. 2*B*								
Rotarod test								
One-way ANOVA followed by		*GPHR^F/+^;GluD2-Cre*			*GPHR^F/F^;GluD2-Cre*			
Sidak’s multiple comoarusins test	Age	Mean	SEM	*n*	Mean	SEM	*n*	Statistics
Time (s)	P45	247.8	± 15.39	5	242.7	± 16.46	5	*p* = 0.9956
	P60	231.0	± 23.67	5	207.5	± 16.56	5	*p* = 0.7158
	P90	226.9	± 16.53	5	146.4	± 12.10	5	*p* = 0.0086
Fig. 2*D*								
Composite phenotype scoring system								
Kruskal–Wallis rank sum test		*GPHR^F/+^;GluD2-Cre*			*GPHR^F/F^;GluD2-Cre*			
followed by Dunn's test	Age	Mean	SEM	*n*	Mean	SEM	*n*	Statistics
Average score	P45	0	0	9	0.222	± 0.147	9	*p* > 0.9999
	P60	0.333	± 0.167	9	0.667	± 0.167	9	*p* > 0.9999
	P90	0.333	± 0.167	9	3.78	± 0.222	9	*p* = 0.0087
	P150	0.222	± 0.147	9	5.667	± 0.236	9	*p* < 0.0001
	P300	0.444	± 0.176	9	6.333	± 0.441	9	*p* = 0.0002
Fig. 3*B*								
Measurement of cerebellar area								
One-way ANOVA followed by		*GPHR^F/+^;GluD2-Cre*			*GPHR^F/F^;GluD2-Cre*			
Sidak’s multiple comoarusins test	Age	Mean	SEM	*n*	Mean	SEM	*n*	Statistics
Area (mm^2^)	P60	7.405	± 0.0449	15	6.883	± 0.0816	15	*p* = 0.0009
	P90	7.340	± 0.0569	15	6.127	± 0.0974	15	*p* < 0.0001
Fig. 3*E*								
Quantitative analysis of PC density								
One-way ANOVA followed by		*GPHR^F/+^;GluD2-Cre*			*GPHR^F/F^;GluD2-Cre*			
Sidak’s multiple comoarusins test	Age	Mean	SEM	*n*	Mean	SEM	*n*	Statistics
PC/µm^2^	P45	0.0150	± 0.00068	15	0.01467	± 0.00052	15	*p* = 0.9213
	P60	0.0147	± 0.00093	15	0.01261	± 0.00049	15	*p* = 0.0002
	P90	0.0158	± 0.00117	15	0.00562	± 0.00120	15	*p* < 0.0001
Related to Fig. 3*F*								
Quantitative analysis of IBA1-positive		*GPHR^F/+^;GluD2-Cre*			*GPHR^F/F^;GluD2-Cre*			
Microglia	Age	Mean	SEM	*n*	Mean	SEM	*n*	Statistics
Unpaired Student’s *t* test	P60	35.69	± 2.70	15	97.730	± 7.86	15	*p* < 0.0001
Microglia/nm^2^								
Related for Fig. 4*A*								
Quantitative analysis of the abnormal		*GPHR^F/F^;GluD2-Cre*						
Golgi in PC	Age	Mean	SEM	*n*				Statistics
Unpaired Student’s *t* test	P45	66.85	± 3.13	15				P45 vs P60
Ratio (abnormal Golgi distribution within	P60	90.58	± 2.23	15				*p* < 0.0001
PC/total number of PCs)								
Related to Fig. 5*A*								
Quantitative analysis of axonal swelling		*GPHR^F/F^;GluD2-Cre*						
in granule cell layer	Age	Mean	SEM	*n*				Statistics
unpaired Student’s *t* test	P45	40.04	± 3.00	15				P45 vs P60
Size of axon swelling (µm^2^)	P60	70.22	± 4.64	15				*p* = 0.0241
		*GPHR^F/F^;GluD2-Cre*						
	Age	Mean	SEM	*n*				Statistics
Density of axon swellings/nm^2^	P45	56.54	± 4.94	15				P45 vs P60
	P60	80.19	± 8.59	15				*p* < 0.0001

Related to Fig. 5*C*								
Quantitative analysis of axonal swelling		*GPHR^F/F^;GluD2-Cre*						
in DCNs	Age	Mean	SEM	*n*				Statistics
Unpaired Student’s *t* test	P45	261.10	± 32.54	15				P45 vs P60
Density of axon swellings/nm^2^	P60	880.60	± 46.07	15				*p* < 0.0001
Related to Fig. 6*A*								
Pinceau structure counting								
One-way ANOVA followed by		*GPHR^F/+^;GluD2-Cre*			*GPHR^F/F^;GluD2-Cre*			
Sidak’s multiple comoarusins test	Age	Mean	SEM	*n*	Mean	SEM	*n*	Statistics
Raito (total number of PC with	P60	89.58	± 1.591	15	17.92	± 1.50	15	*p* < 0.0001
Pinceau structure/total number of PCs)								
		*GPHR^F/+^;GluD2-Cre*			*GPHR^F/F^;L7-Cre*			
	Age	Mean	SEM	*n*	Mean	SEM	*n*	Statistics
	P60	89.58	± 1.591	15	87.46	± 1.50	15	*p* = 0.7015
		*GPHR^FF+^;GluD2-Cre*			*GPHR^F/F^;L7-Cre*			
	Age	Mean	SEM	*n*	Mean	SEM	*n*	Statistics
	P60	17.92	± 1.50	15	87.46	± 1.50	15	*p* < 0.0001
Fig. 7*C*,*D*								
Electrophysiological analysis								
Unpaired Student’s *t* test		*GPHR^F/+^;GluD2-Cre*			*GPHR^F/F^;GluD2-Cre*			
		Mean	SEM	*n*	Mean	SEM	*n*	Statistics
Frequency (Hz)	All	1.340	± 0.1135	4	0.892	± 0.2242	4	*p* = 0.1258
	Small	0.9030	± 0.0460	4	0.727	± 0.1690	4	*p* = 0.3542
	Large	0.4360	± 0.0833	4	0.165	± 0.0680	4	*p* = 0.0454
		Mean	SEM	*n*	Mean	SEM	*n*	Statistics
Amplitude (pA)	All	99.82	± 3.57	4	73.15	± 7.81	4	*p* = 0.0210
	Small	70.00	± 4.97	4	61.16	± 3.99	4	*p* = 0.2147
	Large	162.60	± 19.87	4	138.90	± 6.17	4	*p* = 0.2994
Related to Fig. 8*A*								
vGlut2 positive puncta on PC soma								
One-way ANOVA followed by		*GPHR^F/+^;GluD2-Cre*			*GPHR^F/F^;GluD2-Cre*			
Sidak’s multiple comoarusins test	Age	Mean	SEM	*n*	Mean	SEM	*n*	Statistics
Raito (vGlut2-positive puncta with PC	P60	9.95	± 1.07	15	69.02	± 4.21	15	*p* < 0.0001
somata/total number of PCs)								
		*GPHR^F/+^;GluD2-Cre*			*GPHR^F/F^;L7-Cre*			
	Age	Mean	SEM	*n*	Mean	SEM	*n*	Statistics
	P60	9.95	± 1.07	15	15.06	± 1.300	15	*p* = 0.4383
		*GPHR^F/F^;GluD2-Cre*			*GPHR^F/F^;L7-Cre*			
	Age	Mean	SEM	*n*	Mean	SEM	*n*	Statistics
	P60	69.02	± 4.21	15	15.06	± 1.300	15	*p* < 0.0001
Related to Fig. 8*B*								
mGluR1α-vGlut2 double-positive puncta		*GPHR^F/+^;GluD2-Cre*			*GPHR^F/F^;GluD2-Cre*			
on PC soma	Age	Mean	SEM	*n*	Mean	SEM	*n*	Statistics
Unpaired Student’s *t* test	P60	9.34	± 1.26	15	45.73	± 4.02	15	*p* < 0.0001
Raito (vGlut2-positive puncta with PC								
somata/total number of PCs)								
Related to Fig. 9								
Quantitative analysis of								
lamellar body density		*GPHR^F/+^;GluD2-Cre*			*GPHR^F/F^;GluD2-Cre*			
Unpaired Student’s *t* test	Age	Mean	SEM	*n*	Mean	SEM	*n*	Statistics
Lamellar body/nm	P60	0.29	± 0.06	5	6.49	± 0.55	5	*p* < 0.0001

Movie 1. Lack of motor coordination in *GPHR^F/F^;GluD2-Cre* mice. *GPHR^F/F^;GluD2-Cre* mice display abnormal gait at P90. The control littermates display a normal phenotype. 10.1523/ENEURO.0427-18.2019.video.1

## Results

### Elevated luminal pH of the Golgi apparatus in primary cultured neurons lacking *GPHR*

**Figure 1.**
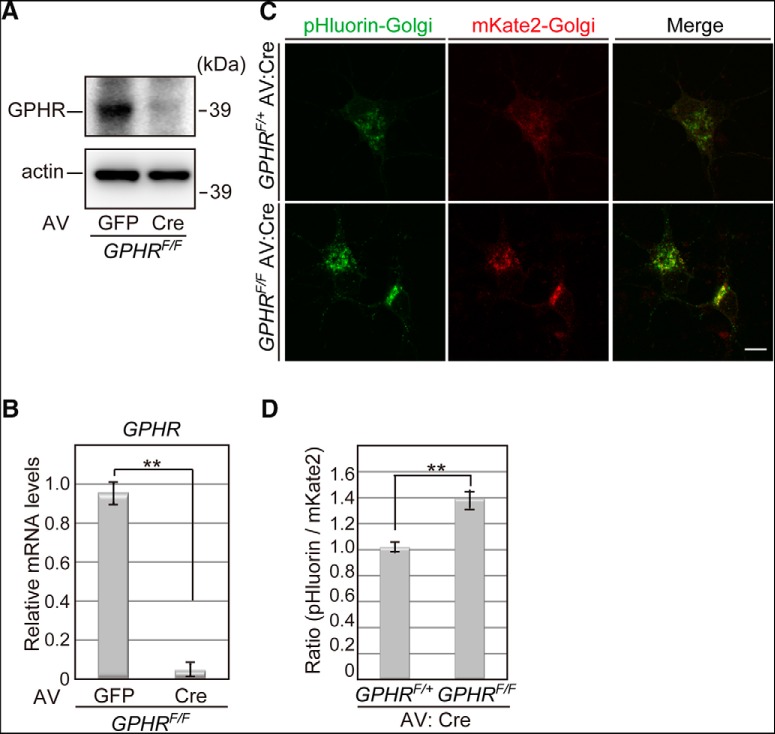
Altered luminal pH of the Golgi apparatus in GPHR deficient primary cultured neurons. ***A***, Immunoblot analysis in primary cultured neurons from *GPHR^F/F^* mice with adenovirus (AV)-based exogenous expression of GFP or Cre recombinase. Nine days after infection, total cell lysates were subjected to immunoblot analysis for GPHR and actin. Data are representative of three independent experiments. ***B***, Quantitation of mRNA levels of *GPHR* in primary cultured neurons manipulated, as described in ***A***. Data are presented as the mean ± SEM; *n* = 6 for each group; **p < 0.01 (unpaired Student’s *t* test). ***C***, Fluorescence images of pHluorin-Golgi in primary cultured neurons from *GPHR^F/+^* and *GPHR^F/F^* mice with AV-based exogenous expression of Cre recombinase. Two days after infection, pHluorin-Golgi-IRES-mKate2-Golgi was transfected. Fluorescence images were taken seven days after transfection. Data are representative of three independent experiments. Scale bar: 20 µm. ***D***, Quantification of the pHluorin/mKate2 fluorescence ratio in primary cultured neurons manipulated as described in ***C***. Data are presented as the mean ± SEM; *n* = 5 for each group; **p < 0.01 (unpaired Student’s *t* test).

Initially, we assessed the disruption of the GPHR gene in primary cultured neuron from *GPHR* floxed embryos by using an adenovirus expression system. Deletion of the GPHR protein was confirmed by immunoblot analysis using an anti-GPHR antibody in primary cultured neurons from GPHR floxed mice expressing Cre recombinase (Fig. 1*A*). As expected, exogenous expression of Cre recombinase suppressed GPHR mRNA levels in primary cultured neurons, as examined by quantitative real-time PCR (Fig. 1*B*; Table 1; *GPHR^F/F^*; AV: GFP: 0.992 ± 0.025, *n* = 6; *GPHR^F/F^*; AV: Cre: 0.044 ± 0.011, *n* = 6; unpaired Student’s *t* test, *t*_(10)_ = 35.41, **p < 0.0001). The function of GPHR for maintaining the luminal pH of the Golgi apparatus in neurons was examined by constructing a signal localized at the Golgi apparatus to the pH sensor protein pHluorin and red fluorescence protein mKate2, respectively (Miesenböck et al., 1998; Tanida et al., 2014). Using the constructed tandem expression plasmid (i.e., pHluorin-Golgi-IRES-mKate2-Golgi), alkalization of the Golgi apparatus lumen in response to deletion of GPHR in primary cultured neurons was monitored. The fluorescence intensity of pHulorin markedly increased, indicating that deletion of GPHR elevated the luminal pH of the Golgi apparatus in primary cultured neurons (Fig. 1*C*). Quantitative measurements confirmed that the pHluorin/mKate2 fluorescent ratio of primary cultured neurons lacking *GPHR* (*GPHR^F/F^*; AV: Cre) is higher than that of the control (*GPHR^F/+^*; AV: Cre) neurons (Fig. 1*D*; Table 1; *GPHR^F/+^*; AV: Cre: 1.015 ± 0.038, *n* = 5; *GPHR^F/F^*; AV: Cre: 1.390 ± 0.068, *n* = 5; unpaired Student’s *t* test, *t*_(8)_ = 4.827, **p = 0.0013). These results suggest that GPHR is responsible for maintaining normal luminal acidic conditions of the Golgi apparatus in neurons.

### *GPHR^F/F^;GluD2-Cre* mice show cerebellar ataxia with progressive neurodegeneration

**Figure 2.**
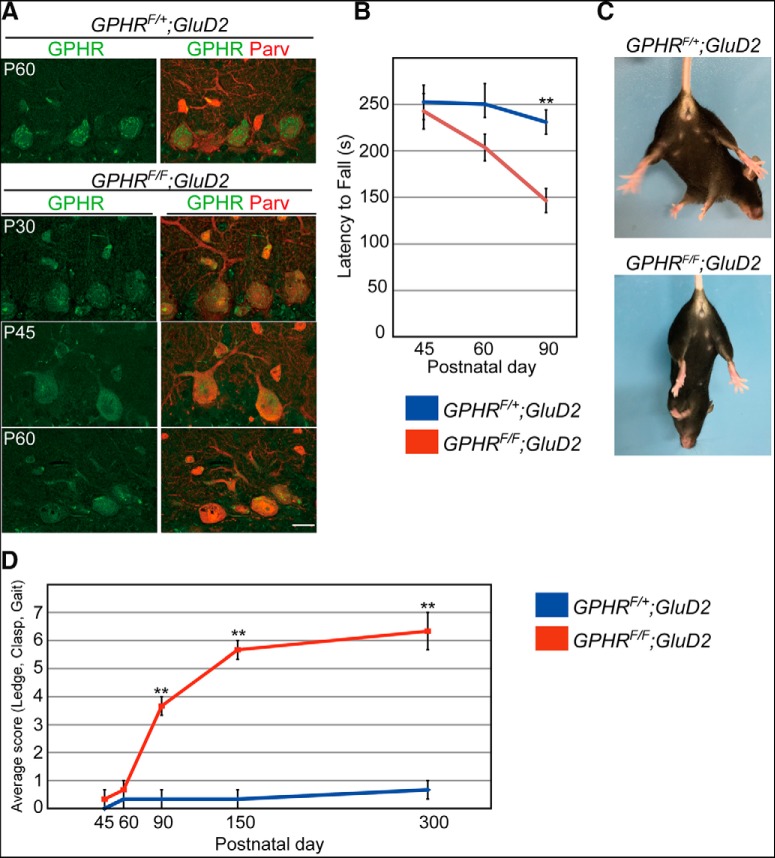
*GPHR^F/F^;GluD2-Cre* mice lack motor coordination. ***A***, GPHR is deleted in PCs. Double immunofluorescence for GPHR (green) and parvalbumin (Parv; red) in the PC layer from *GPHR^F/+^;GluD2-Cre* mice at P60 and *GPHR^F/F^;GluD2-Cre* mice at P30, P45, and P60. Dotted immunoreactivity for GPHR is not discernable in PCs from *GPHR^F/F^;GluD2-Cre* mice after P45. Scale bars: 20 µm. ***B***, Accelerating rotarod test for *GPHR^F/+^;GluD2-Cre* and *GPHR^F/F^;GluD2-Cre* mice. The average retention time on the rotarod is shown (*n* = 5 mice, female); **p < 0.001 (one-way ANOVA with Sidak’s *post hoc* test). ***C***, Limb clasping reflex in *GPHR^F/F^;GluD2-Cre* mice at P90. When lifted by the tail, *GPHR^F/+^;GluD2-Cre* mice extended their hind limbs, whereas *GPHR^F/F^;GluD2-Cre* mice moved their legs to the trunk. ***D***, Behavioral analyses of *GPHR^F/F^;GluD2-Cre* mice relative to *GPHR^F/+^;GluD2-Cre* mice. Graph showing cerebellar ataxic phenotypes score of *GPHR^F/+^;GluD2-Cre* and *GPHR^F/F^;GluD2-Cre* mice at P45, P60, P90, P150, and P300 (three mice per group). Average composite score for each genotype at each age was calculated. Data are presented as the mean ± SEM; *n* = 9 trial for each group, **p < 0.01 (Kruskal–Wallis test followed by the Dunn’s test). For ***A***, three mice per genotype; *n* = 5 sections per mouse; representative images are shown. See also Movie 1.

Next, to study the function of GPHR in neurons *in vivo*, we generated PC- and BC-specific GPHR knock-out mice by crossing *GPHR* floxed mice with *GluD2-Cre* mice, in which Cre recombinase was knocked-in into the glutamate receptor δ2 (GluD2) locus (Yamasaki et al., 2011). Cre-mediated deletion of GPHR in PCs was completed by postnatal day (P) 45 (Fig. 2*A*). Results from the rotarod test showed that *GPHR^F/F^; GluD2-Cre* mice showed uncoordinated movement from P90 (Fig. 2*B*; Table 1; one-way ANOVA, *n* = 5 mice for each group, female; *F*_(5,24)_ = 4.753, *p* = 0.0037; and Sidak’s *post hoc* test, control vs *GPHR^F/F^;GluD2-Cre*, *p* = 0.9956 at P45, *p* = 0.7158 at P60 and **p = 0.0086 at P90). Composite phenotype scoring of the hindlimb clasping, open field gait and ledge tests (Guyenet et al., 2010) confirmed that *GPHR^F/F^;GluD2-Cre* mice older than P90 displayed ataxic phenotypes as judged by loss of gait coordination and balance, reduced locomotor activity and abnormal hindlimb clasping, which are commonly observed in mice with cerebellar degeneration (Fig. 2*C*,*D*; Movie 1; Table 1; Kruskal–Wallis test, three mice per group; three trials per mouse; *n* = 9 trials for each group, KS = 69.33, *p* < 0.0001; Dunn’s *post hoc* test, control vs *GPHR^F/F^;GluD2-Cre*, *p* > 0.9999 at P45, *p* > 0.9999 at P60, **p = 0.0087 at P90, **p < 0.0001 at P150, **p = 0.0002 at P300). These phenotypes indicated that deletion of *GPHR* in PCs and BCs leads to cerebellar ataxia.

**Figure 3.**
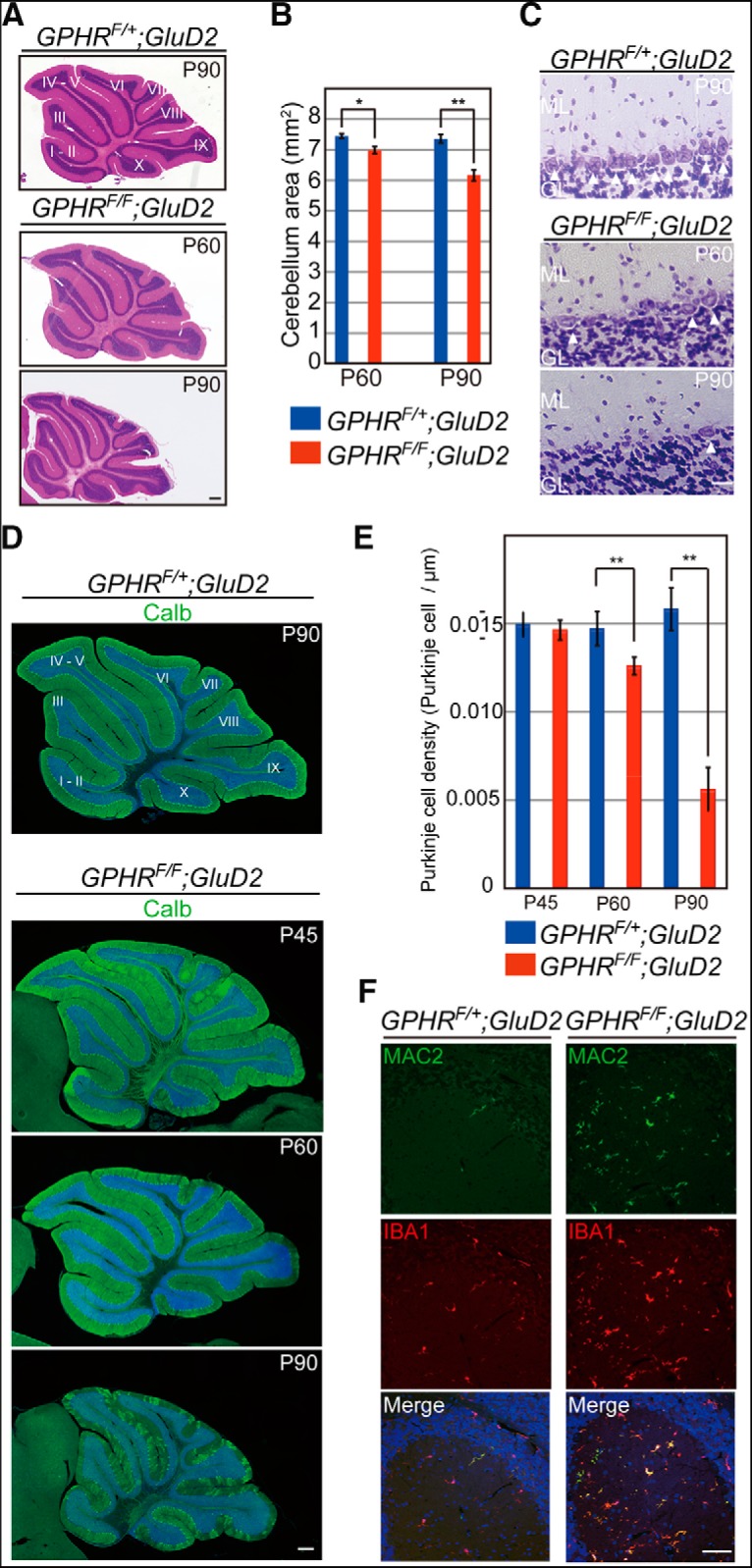
Progressive neurodegeneration in *GPHR^F/F^;GluD2-Cre* mice. ***A***, H & E-stained images of sagittal sections from *GPHR^F/+^;GluD2-Cre* mice at P90 and *GPHR^F/F^;GluD2-Cre* mice at P60 and P90. Cerebellar lobules are indicated by Roman numerals. Scale bar: 300 µm. ***B***, Measurement of cerebellar areas in *GPHR^F/+^;GluD2-Cre* and *GPHR^F/F^;GluD2-Cre* mice at P60 and P90 (three mice per group). Five sections from each mouse corresponding to the cerebellar region, as shown in ***A***, were measured. Data are presented as the mean ± SEM; *n* = 15 sections for each group, *p < 0.05, **p < 0.01 (one-way ANOVA with Sidak’s *post hoc* test). ***C***, Nissl staining of the PC layer of lobule IX in the cerebellum from *GPHR^F/+^;GluD2-Cre* mice at P90 and *GPHR^F/F^;GluD2-Cre* mice at P60 and P90. Arrowheads indicate PCs. ML, molecular layer; GL, granule cell layer. Scale bar: 30 µm. ***D***, Immunofluorescence for calbindin (Calb; green) in the cerebellum of *GPHR^F/+^;GluD2-Cre* mice at P90 and *GPHR^F/F^;GluD2-Cre* mice at P45, P60, and P90. Nuclei are stained with DAPI (blue). Cerebellar lobules are indicated by Roman numerals. Scale bar: 300 µm. ***E***, The density of PCs was measured following immunofluorescence staining for calbindin *GPHR^F/+^;GluD2-Cre* and *GPHR^F/F^;GluD2-Cre* mice at P45, P60, and P90. Five sections from each mouse corresponding to the cerebellar region as shown in ***D*** were measured. Data are presented as the mean ± SEM; *n* = 15 sections for each group; **p < 0.01 (one-way ANOVA with Sidak’s *post hoc* test). ***F***, Double immunofluorescence for IBA1 (green) and MAC2 (red) in the cerebellum of *GPHR^F/+^;GluD2-Cre* and *GPHR^F/F^;GluD2-Cre* mice at P60. Nuclei are stained with DAPI (blue). Scale bar: 50 µm. For ***A***, ***C***, ***D***, ***F***, three mice per genotype; *n* = 5 sections per mouse; representative images are shown.

H & E staining at P60 revealed difference in the size of the cerebellum between control and *GPHR^F/F^;GluD2-Cre* mice, and at P90 the difference was more pronounced (Fig. 3*A*,*B*; Table 1; one-way ANOVA, three mice per group; five sections per mouse; *n* = 15 section for each group, *F*_(3,56)_ = 42.26, *p* < 0.0001; and Sidak’s *post hoc* test, control vs *GPHR^F/F^;GluD2-Cre*, **p = 0.003 at P60, control vs *GPHR^F/F^;GluD2-Cre*, **p < 0.0001 at P90). The histological feature of PC lost was detected in *GPHR^F/F^;GluD2-Cre* mice at P60 and was more severe at P90, as examined by Nissl staining (Fig. 3*C*). Consistent with Nissl staining, loss of PCs was also detected by calbindin immunostaining. *GPHR^F/F^;GluD2-Cre* mice exhibited loss of PCs especially in lobule IX at P60 and a decrease in PC density was observed in whole cerebellar lobules at P90, as examined by calbindin immunostaining (Fig. 3*D*,*E*; Table 1; one-way ANOVA, three mice per group; five sections per mouse; *n* = 15 sections for each group, *F*_(5,96)_ = 112.2, *p* < 0.0001; and Sidak’s *post hoc* test, control vs *GPHR^F/F^;GluD2-Cre*, *p* = 0.9213 at P45, **p = 0.002 at P60, **p < 0.0001 at P90). The number of PCs in control and *GPHR^F/F^;GluD2-Cre* mice by stereological methods were estimated to confirm these histological results (Table 2). Comparison of the number of PCs in the cerebellum between control and *GPHR^F/F^;GluD2-Cre* mice at P90 revealed that there were only ∼20% of the PCs remaining in *GPHR^F/F^;GluD2-Cre* mice. These results suggest that the number of PCs in *GPHR^F/F^;GluD2-Cre* mice decreases in an age-dependent manner. Corresponding to the degenerative changes in PCs, activated microglia immunopositive for IBA1 and/or MAC2 were abundant in the cerebellum of *GPHR^F/F^;GluD2-Cre* mice (Fig. 3*F*). Quantification confirmed that the density of IBA1-immunopositive microglia in lobule IX increased significantly in *GPHR^F/F^;GluD2-Cre* mice when compared with that of control mice (Table 1; *GPHR^F/+^;GluD2-Cre*: 35.69 ± 2.706; *GPHR^F/F^;GluD2-Cre*: 97.73 ± 7.861, three mice per group; five sections per mouse; *n* = 15 sections for each group, unpaired Student’s *t* test, *t*_(28)_ = 7.463, **p < 0.0001).

**Table 2.** Stereological estimates of total number of the cerebellar PCs (values are in thousands)

	Animal	*GPHR^F/+^;GluD2-Cre*	*GPHR^F/F^;GluD2-Cre*
Age		PC number	PC number
P45 #1		161.62	159.89
P45 #2		182.74	166.71
P45 #3		198.85	190.07
	Mean	181.07	172.22
	SEM	± 10.78	± 9.14
			
Age		PC number	PC number
P60 #1		161.01	133.28
P60 #2		166.29	136.22
P60 #3		190.51	144.99
	Mean	172.60	138.16
	SEM	± 9.08	± 3.52
			
Age		PC number	PC number
P90 #1		158.59	28.16
P90 #2		171.45	28.31
P90 #3		182.81	36.94
	Mean	170.95	31.14
	SEM	± 7.00	± 2.90

### GPHR loss causes abnormal morphology of the Golgi apparatus

**Figure 4.**
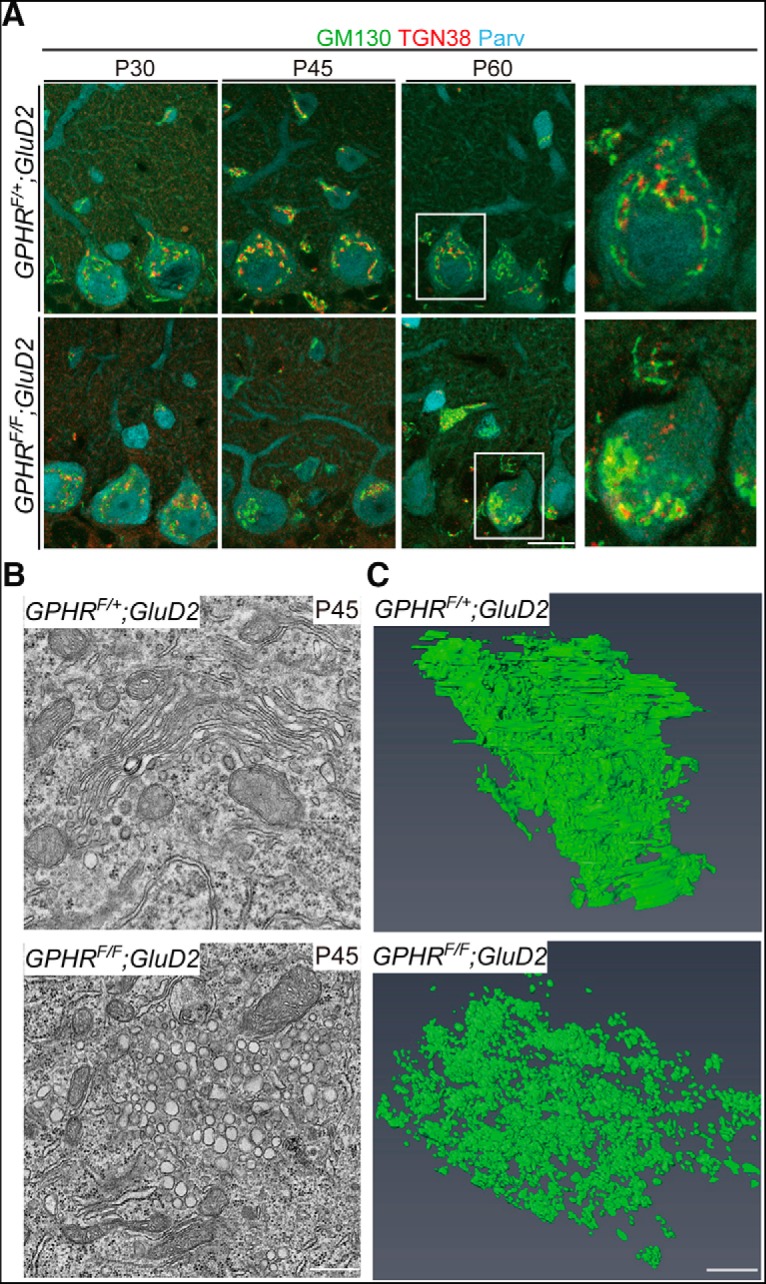
Deletion of *GPHR* has an effect on the Golgi apparatus organization and distribution in PCs. ***A***, Triple immunofluorescence for GM130 (cis-Golgi; green), TGN38 (trans-Golgi; red) and parvalbumin (Parv; cyan) in PCs from *GPHR^F/+^;GluD2-Cre* and *GPHR^F/F^;GluD2-Cre* mice at P30, P45, and P60. Scale bar: 20 µm. ***B***, Ultrastructural analysis of Golgi apparatus structures in PC somata from *GPHR^F/+^;GluD2-Cre* and *GPHR^F/F^;GluD2-Cre* mice at P45. Typical Golgi apparatus structures with flattened cisterns in *GPHR^F/+^;GluD2-Cre* mice were distinguishable from the Golgi apparatus structures with clusters of vacuoles in *GPHR^F/F^;GluD2-Cre* mice. ***C***, The Golgi apparatus structure reconstructed from serial FIB/SEM images in PCs from *GPHR^F/+^;GluD2-Cre* and *GPHR^F/F^;GluD2-Cre* mice at P45. 3D model shows that fragmentation of the Golgi apparatus occurred in PCs from *GPHR^F/F^;GluD2-Cre* mice. Scale bars: 500 nm. For ***A***, ***B***, three mice per genotype were used; *n* = 5 sections per mouse; representative images are shown. See also Movies 1, 2.

Alkalization of the Golgi luminal pH causes morphological abnormalities of the Golgi apparatus in cultured cells (Tartakoff, 1983; Sakaguchi et al., 1996; Maeda et al., 2008). However, very little information can be gleaned from *in vivo* literature. In normal PCs, the Golgi apparatus is properly distributed by forming a perinuclear strand (Palay and Chan-Palay, 1974; Liu et al., 2017). To characterize the Golgi apparatus in PCs of *GPHR^F/F^;GluD2-Cre* mice, we performed double staining for GM130 and TGN38, which are markers for *cis-* and *trans*-Golgi, respectively. In control PCs, GM130- and TGN38-positive Golgi signals were distributed as perinuclear small patches, which were enriched at the apical pole facing the primary dendrite. Each Golgi stack was designated as *cis*- and *trans*-cisterns. In contrast, GPHR deficiency caused perinuclear accumulation of the immunopositive areas and some were shifted to the basolateral perikarya at P45, indicating an abnormal distribution of the Golgi apparatus (Fig. 4*A*). After P45, the Golgi apparatus appeared as a mixture of GM130- and TGN38-positive areas (Fig. 4*A*). Quantification confirmed that the ratio of the abnormal Golgi apparatus distribution in PCs of *GPHR^F/F^;GluD2-Cre* mice increased significantly in an age-dependent manner (Table 1; *GPHR^F/F^;GluD2-Cre* at P45: 66.85 ± 3.13; *GPHR^F/F^;GluD2-Cre* at P60: 90.58 ± 2.23, three mice per group; five sections per mouse; *n* = 15 sections for each group; *GPHR^F/F^;GluD2-Cre* at P45: 185 PCs; *GPHR^F/F^;GluD2-Cre* at P60: 163 PCs; unpaired Student’s *t* test, *t*_(28)_ = 6.174, **p < 0.0001). EM showed that instead of stacks of flattened cisterns, clusters of vacuolar structures were observed in *GPHR*-deficient PCs at P45, suggesting fragmentation of the Golgi apparatus (Fig. 4*B*). Furthermore, volumetric reconstruction of the Golgi apparatus by FIB-SEM confirmed fragmentation of the Golgi apparatus with clusters of vacuoles in PCs from *GPHR^F/F^;GluD2-Cre* mice (Fig. 4*C*; Movies 2, 3). These data indicated that acidification of the luminal pH of the Golgi apparatus is important for maintaining Golgi apparatus morphology and distribution in PCs.

Movie 2. Animation of the three-dimensional reconstructed structure of the Golgi apparatus in a PC of *GPHR^F/+^;GluD2-Cre* mice at P45. This reconstructed image is obtained from the segmented volume of the serial FIB/SEM images and corresponds to Figure 2*C*, upper panel. Cisternae of the Golgi apparatus and the nucleus are shown by green and magenta, respectively. 10.1523/ENEURO.0427-18.2019.video.2

Movie 3. Animation of the three-dimensional reconstructed abnormal structure of the Golgi apparatus in a PC of *GPHR^F/F^;GluD2-Cre* mice at P45. This reconstructed image is obtained from the segmented volume of the serial FIB/SEM images and corresponds to Figure 2*C*, lower panel. Vesiculated structures of the Golgi apparatus and the nucleus are shown by green and magenta, respectively. 10.1523/ENEURO.0427-18.2019.video.3

### Golgi abnormality is accompanied by axonal and synaptic impairment in PCs

**Figure 5.**
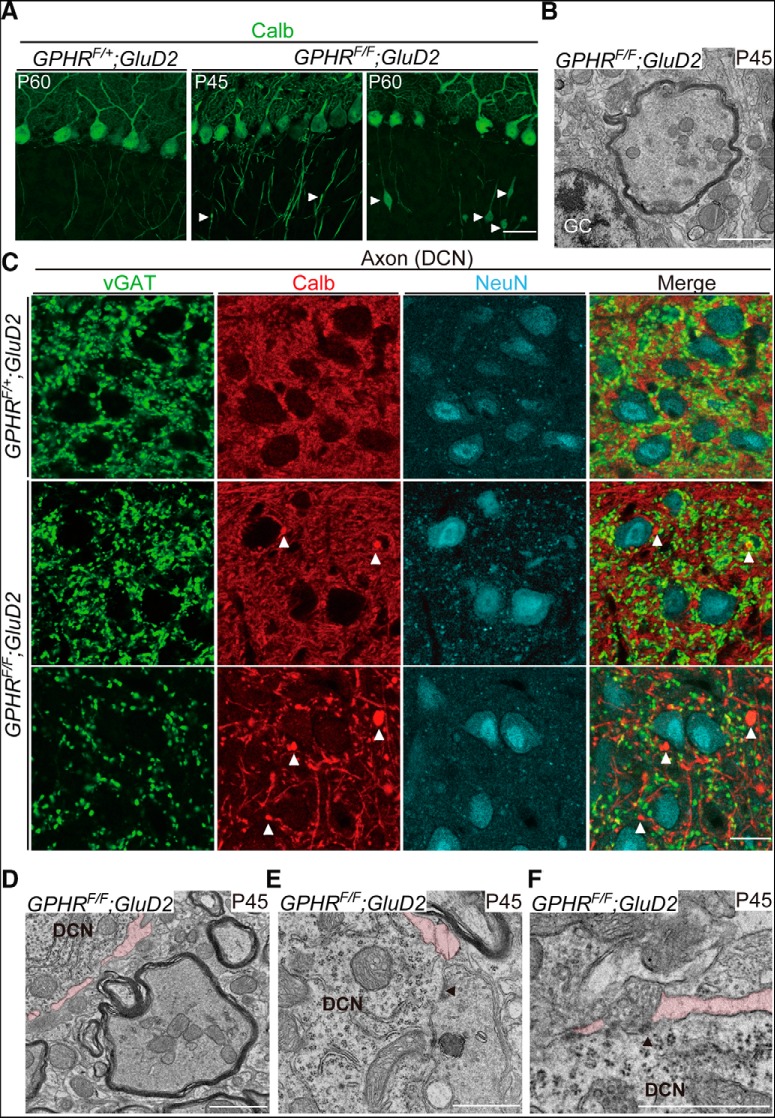
Axonal swelling and abnormal synaptic terminal formation in *GPHR*-deficient PCs. ***A***, Immunofluorescence for calbindin (Calb; green) in the PC layer of *GPHR^F/+^;GluD2-Cre* mice at P60 and *GPHR^F/F^;GluD2-Cre* mice at P45 and P60. Arrowheads indicate axon swellings. Scale bar: 20 µm. ***B***, Electron micrographs show axon swelling in the granule cell layer at P45. GC, granule cell. Scale bar: 1 µm. ***C***, Triple immunofluorescence for vGAT (green), calbindin (Calb; red), and NeuN (cyan) in the DCN of *GPHR^F/+^;GluD2-Cre* mice at P60 and *GPHR^F/F^;GluD2-Cre* mice at P45 and P60. PC axon terminal swelling in the DCNs of *GPHR^F/F^;GluD2-Cre* mice. Arrowheads indicate swellings in the distal axons or axon terminals positive for calbindin. Scale bar: 20 µm. ***D***−***F***, Electron micrographs showing an axonal swelling in the DCN region (***D***), an abnormal synaptic terminal (***E***), and intercellular spaces (highlighted by pink) on and around the DCN neuron, respectively, at P45. An arrowhead indicates the active zone. Scale bars: 1 µm. For ***A****−****F***, three mice per genotype were used; *n* = 5 sections per mouse; representative images are shown.

We reported previously that the regulation of luminal pH of the Golgi apparatus is indispensable for ensuring the normal function of the Golgi apparatus, such as glycosylation and vesicular transport in cultured cells (Maeda et al., 2008). In addition, the Golgi apparatus is important for axonal transport of neurotransmitters in mature neurons (Campenot et al., 2003; Horton et al., 2005; Matsuki et al., 2010; Liu et al., 2017). Thus, morphological alterations in PC axons on perturbations in luminal pH of the Golgi apparatus were investigated. PC axons traverse the granule cell layer to innervate neurons in DCN. First, we focused on PC axons in the granule cell layer. Different from control axons at P60 (Fig. 5*A*), calbindin immunostaining revealed that axon swellings appeared at P45 in *GPHR^F/F^;GluD2-Cre* mice, and these areas increased in size and number at P60 (Table 1; size: *GPHR^F/F^;GluD2-Cre* at P45: 40.04 ± 2.99 µm^2^; *GPHR^F/F^;GluD2-Cre* at P60: 70.22 ± 4.63 µm^2^, unpaired Student’s *t* test, *t*_(28)_ = 6.405, **p < 0.0001; density of *GPHR^F/F^;GluD2-Cre* mice at P45: 56.54 ± 4.94/nm^2^; *GPHR^F/F^;GluD2-Cre* at P60: 80.19 ± 8.56/nm^2^, unpaired Student’s *t* test, *t*_(28)_ = 2.384, *p = 0.0241; three mice per group; five sections per mouse; *n* = 15 sections for each group). EM observations revealed myelinated swelling axons in the granular cell layer at P45 in *GPHR^F/F^;GluD2-Cre* mice (Fig. 5*B*). In the DCN region of *GPHR^F/F^;GluD2-Cre* mice, swelling axons also appeared at P45 and increased prominently in number at P60 (Fig. 5*C*; Table 1; density of *GPHR^F/F^;GluD2-Cre* mice at P45: 261.1 ± 32.54/nm^2^; *GPHR^F/F^;GluD2-Cre* at P60: 880.6 ± 46.07/nm^2^, three mice per group; five sections per mouse; *n* = 15 sections for each group, unpaired Student’s *t* test, *t*_(28)_ = 10.98, *p < 0.0001). Many of these swelling axons were positive for calbindin but negative for the vGAT, which would reflect swelling axons containing materials other than synaptic vesicles, as shown in the EM observations (Fig. 5*C*,*D*). Interestingly, some presynaptic PC terminals exhibited an abnormal ultrastructural profile and showed symmetrical synaptic contacts with electron-dense postsynaptic density (Fig. 5*E*). In addition, we found that axosomatic synapses on individual DCN neurons were reduced as early as P45, leading to intercellular spaces being created around the DCN neurons (Fig. 5*D–F*). These results indicated that the impairment of acidic conditions in the Golgi apparatus causes axonal and synaptic abnormalities in PCs.

### GPHR is required for maintaining the Pinceau structure

**Figure 6.**
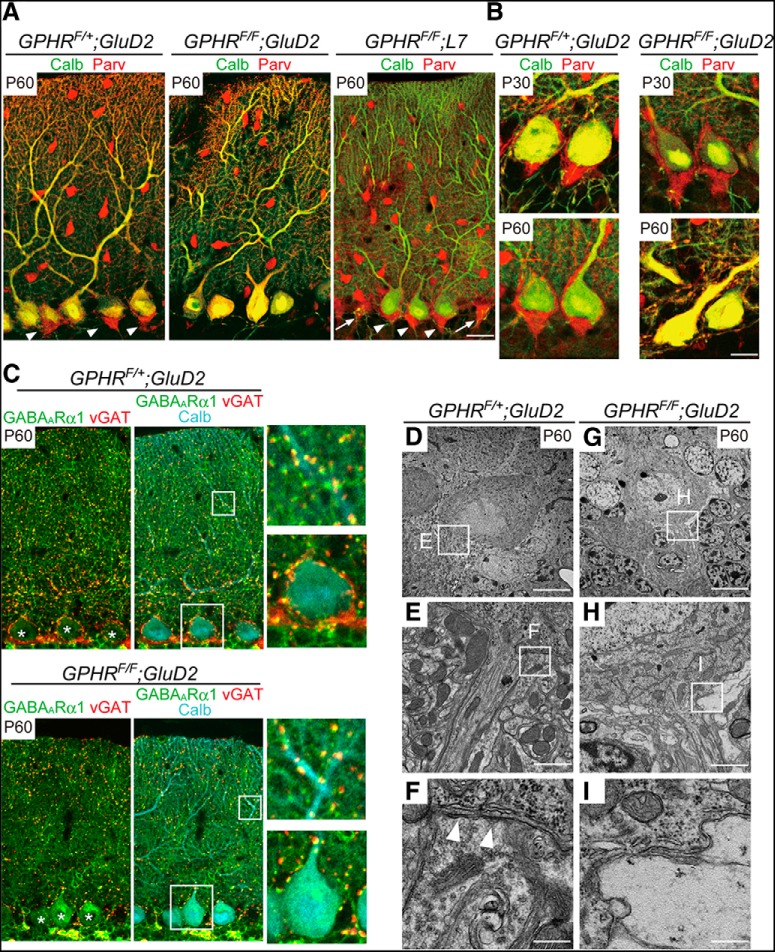
Disappearance of Pinceau structures in *GPHR^F/F^;GluD2-Cre* mice. ***A***, Double immunofluorescence for calbindin (Calb; green) and parvalbumin (Parv; red) in the PC layer from *GPHR^F/+^;GluD2-Cre*, *GPHR^F/F^;GluD2-Cre*, and *GPHR^F/F^;L7-Cre* mice at P60. Arrowheads indicate Pinceau structures. Arrows indicate Pinceau structures without PCs. Scale bar: 20 µm. ***B***, Higher magnification images of Pinceau structures. At P30, the Pinceau structures form cone-shaped structures in both *GPHR^F/+^;GluD2-Cre* and *GPHR^F/F^;GluD2-Cre* mice, whereas this structure is absent at P60 for *GPHR^F/F^;GluD2-Cre* mice. Scale bar: 20 µm. ***C***, Triple immunofluorescence for GABA_A_ Rα1 (green), vGAT (red) and calbindin (cyan) of the molecular layer at P60. GABA_A_ Rα1 and vGAT colocalized on PC somata and dendrites in *GPHR^F/+^;GluD2-Cre* mice, whereas it decreased in PC somata, but not in the distal dendrites of *GPHR^F/F^;GluD2-Cre* mice. Boxed regions are enlargements and presented in the right panels. Asterisks indicate PC somata. Scale bar: 20 µm. Electron micrographs of PC soma (***D***, ***G***) and AIS (***E***, ***F***, ***H***, ***I***) in *GPHR^F/+^;GluD2-Cre* (***D−F***) and *GPHR^F/F^;GluD2-Cre* mice (***G−I***) at P60. Boxed regions in ***D***, ***G*** are enlarged in ***E***, ***H***, respectively. Boxed regions in ***E***, ***H*** are enlarged in ***F***, ***I***, respectively. Disruption of BC axon terminal and Bergman glial processes in the Pinceau area of *GPHR^F/F^;GluD2-Cre* mice (***D****–****F***). Scale bars: 5 µm (***A***, ***D***), 1 µm (***B***, ***E***), 500 nm (***C***, ***F***). Arrowheads indicate an active zone. For ***A****−****I***, three mice per genotype were used; *n* = 5 sections per mouse for each genotype; representative images are shown.

Next, we investigated the innervation of BCs, one of the GABAergic interneurons, to the somatodendritic compartment of PCs. The axons of BCs extend toward the bottom of the PC soma and their branches are densely terminated around the axon initial segment (AIS), forming a structure known as the Pinceau (Palay and Chan-Palay, 1974; Gianola et al., 2003; Ango et al., 2004; Sotelo, 2008). We performed double immunostaining of parvalbumin, which labels both PCs and GABAergic interneurons in the cerebellar molecular layer and calbindin that selectively labels PCs. While control BC axons completely surrounded the PC somata forming Pinceau structures around AIS at P60, *GPHR^F/F^;GluD2-Cre* mice exhibited markedly lower numbers of Pinceau structures at P60 (Fig. 6*A*). To confirm whether the loss of Pinceau structures is because of GPHR-deficiency in BCs, we further analyzed this phenomenon using the PC-specific *GPHR* knock-out mice, *GPHR^F/F^;L7-Cre* mice. Interestingly, the Pinceau structures remained intact in *GPHR^F/F^;L7-Cre* mice at P60 and even after the loss of PCs (Fig. 6*A*, arrows). Quantification confirmed that the mean Pinceau frequency was significantly reduced in *GPHR^F/F^;GluD2-Cre* mice when compared with that of control and *GPHR^F/F^;L7-Cre* mice (Table 1; control: 89.58 ± 1.59%; *GPHR^F/F^;GluD2-Cre*: 17.92 ± 1.50%; *GPHR^F/F^;L7-Cre*: 87.46 ± 1.50%, three mice per group; five sections per mouse; *n* = 15 sections for each group; *GPHR^F/+^;GluD2-Cre*: 203 PCs; *GPHR^F/F^; GluD2-Cre*: 156 PCs; *GPHR^F/F^; L7-Cre*: 160 PCs; one-way ANOVA, *F*_(2,42)_ = 710.4, *p* < 0.0001; and Sidak’s *post hoc* test, control vs *GPHR^F/F^;GluD2-Cre*, **p < 0.0001, control vs *GPHR^F/F^;L7-Cre*, *p* = 0.7015, *GPHR^F/F^;GluD2-Cre* vs *GPHR^F/F^;L7-Cre*, **p < 0.0001). These data suggest that the physical integrity of Pinceau structures depends strongly on the axon status of BCs. Moreover, in *GPHR^F/F^;GluD2-Cre* mice, Pinceau structures were still observed at P30, whereas they were lost at P60 (Fig. 6*B*), indicating that Pinceau structures were formed until stabilization of cerebellar circuits.

Double immunostaining for the GABA_A_ Rα1 and vGAT to label postsynapses and presynapses, respectively, was performed to investigate whether BC axons in *GPHR^F/F^;GluD2-Cre* mice still form synapses with PC soma at P60. GABA_A_ Rα1- and vGAT-immunopositive punctae overlapped almost completely along the soma of PCs in control mice at P60, displaying the typical pattern of BC axon terminals (Fig. 6*C*). In contrast, punctae that were doubly positive for GABA_A_ Rα1 and vGAT were only sporadically detected in PC somata of *GPHR^F/F^;GluD2-Cre* mice (Fig. 6*C*). Unlike BCs, there were no apparent changes in the distribution of stellate cell axon terminals (Fig. 6*C*). We further performed EM analysis to determine the ultrastructural changes in the AIS and soma of PCs of *GPHR^F/F^;GluD2-Cre* mice. In control mice, BC axons formed synaptic contacts on the surfaces of PC soma with typical ultrastructural features of asymmetrical synapses, such as synaptic vesicle accumulation in presynaptic terminals, rigid alignment of pre- and postsynaptic partners and electron dense postsynaptic membranes (Fig. 6*D–F*). However, the PC soma of the *GPHR^F/F^;GluD2-Cre* mice was predominantly enwrapped with electron-lucent processes of Bergmann glia (BG) instead of synaptic contacts with BCs, suggesting that BG processes filled the space caused by BC degeneration (Fig. 6*G–I*).

**Figure 7.**
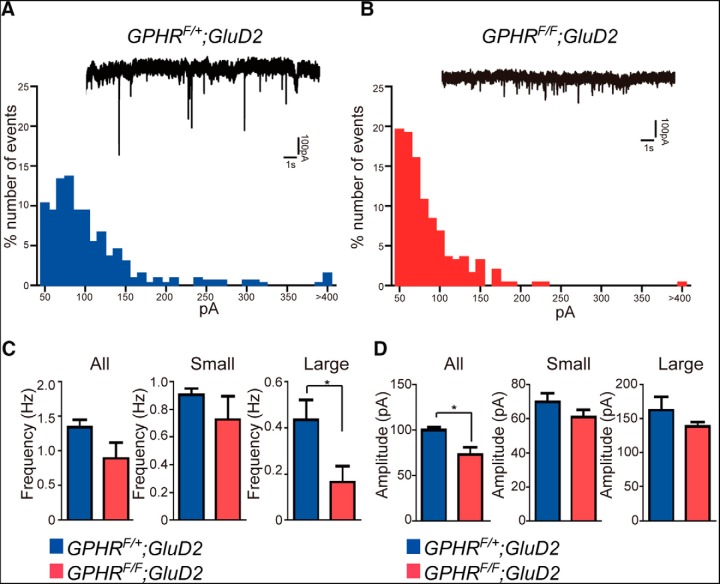
The alteration in GABAergic transmission in PCs from *GPHR^F/F^;GluD2-Cre* mice. Histogram showing the distribution of mIPSC amplitude in *GPHR^F/+^;GluD2-Cre* mice (***A***) and *GPHR^F/F^;GluD2-Cre* mice (***B***) PCs. ACSF containing 1 µM TTX and 10 µM CNQX was used. Insets are representative mIPSC traces. Holding potential (Vh) = −70 mV. ***C***, The bar graphs show the average frequency of mIPSC for all, small (<100 pA), and large (>100 pA) events in *GPHR^F/+^;GluD2-Cre* mice (*n* = 4) and *GPHR^F/F^;GluD2-Cre* mice (*n* = 4). Data are presented as the mean ± SEM; *p < 0.05 (unpaired Student’s *t* test). ***D***, The bar graphs show the average amplitude of mIPSC for all, small (<100 pA), and large (>100 pA) events in *GPHR^F/+^;GluD2-Cre* mice (*n* = 4) and *GPHR^F/F^;GluD2-Cre* mice (*n* = 4). Data are presented as the mean ± SEM; *p < 0.05 (unpaired Student’s *t* test).

To further examine whether the elimination of BC contacts at PCs is concomitantly accompanied by a decrease in synaptic transmission, we recorded mIPSCs from PCs as a readout of the neurotransmission from BCs. mIPSCs in PCs have been previously classified into two types: small mIPSCs from SCs and large mIPSCs from BCs (Nakayama et al., 2012). The occurrence of large amplitude mIPSCs was dramatically reduced in *GPHR^F/F^;GluD2-Cre* mice (*n* = 4) when compared with that of control mice (*n* = 4; Fig. 7*A*,*B*). Accordingly, the mean frequency of large mIPSCs in *GPHR^F/F^;GluD2-Cre* mice was also significantly lower than that of control mice (Fig. 7*C*; Table 1; control: 0.44 ± 0.08 Hz, *n* = 4; *GPHR^F/F^;GluD2-Cre*: 0.17 ± 0.07 Hz, *n* = 4; unpaired Student’s *t* test, *t*_(6)_ = 2.518, *p = 0.0454). Furthermore, the mean amplitude of whole mIPSCs was reduced slightly in *GPHR^F/F^;GluD2-Cre* mice (Fig. 7*D*; Table 1; control: 99.9 ± 3.6 pA, *n* = 4; *GPHR^F/F^;GluD2-Cre*: 73.2 ± 7.8 pA, *n* = 4; unpaired Student’s *t* test, *t*_(6)_ = 3.105, *p = 0.021). In contrast, the mean frequencies of small and large amplitude mIPSCs were not different, indicating that the specific reduction of large amplitude events was due to the disruption of BC axons from the PC soma. Taken together, these results indicated that BC synapses on PCs were dramatically reduced, due largely to the axonal degeneration of BCs caused by the dysfunction of Golgi apparatus in *GPHR^F/F^;GluD2-Cre* mice.

### Formation of excitatory synapses on PC soma in *GPHR^F/F^;GluD2-Cre* mice

**Figure 8.**
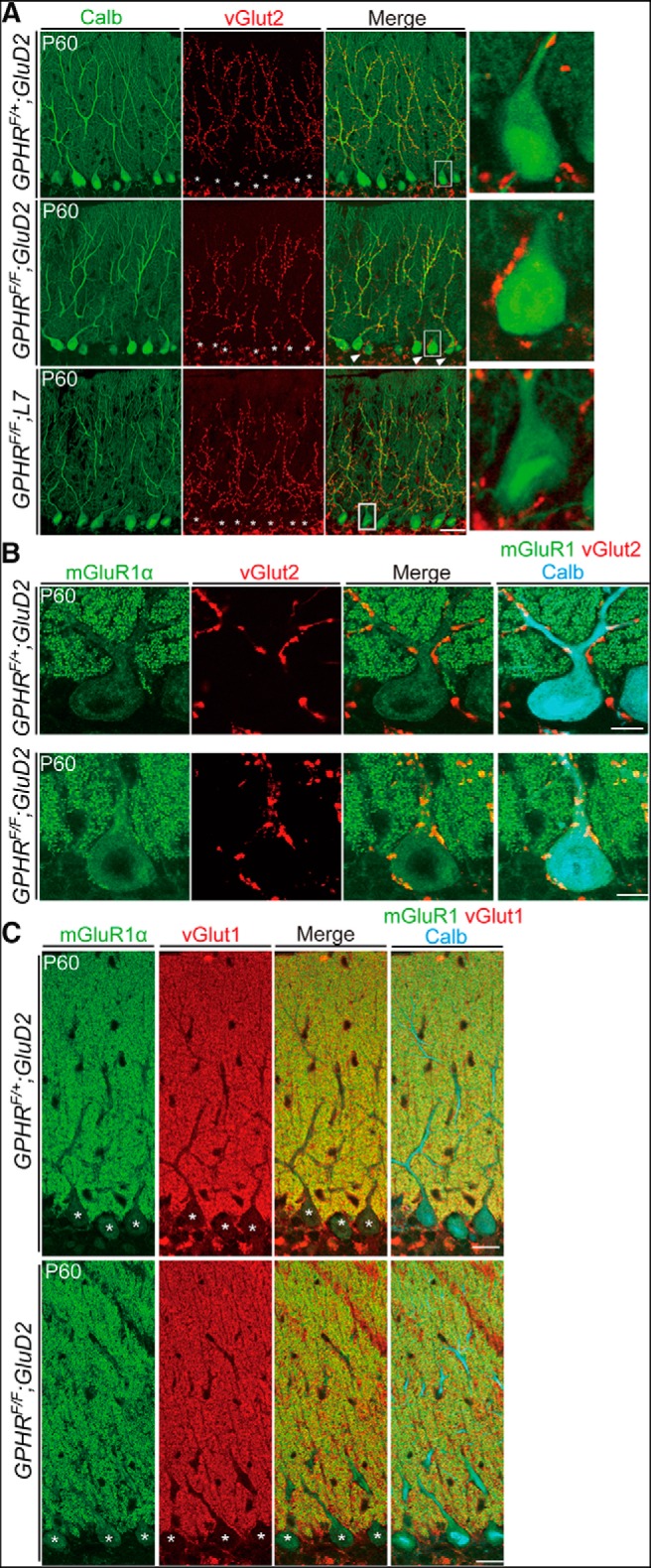
CF terminals innervate PC somata in *GPHR^F/F^;GluD2-Cre* mice. ***A***, Double immunofluorescence for calbindin (Calb; green) and vGlut2 (red) in the molecular layer at P60. vGlut2-positive CF terminals were observed on PC somata in addition to the dendrites of *GPHR^F/F^;GluD2-Cre* mice. Arrowheads indicate vGlut2-positive CF terminals on PC soma. Scale bars: 50 µm. Boxed regions are enlarged and shown in the right panels. ***B***, Triple immunofluorescence for mGluR1α (green), vGlut2 (red), and calbindin (Calb; cyan) in PCs at P60. New spines are formed on the surfaces of PC somata in *GPHR^F/F^;GluD2-Cre* mice. Asterisks indicate PC somata. Scale bars: 10 µm. ***C***, Distribution of parallel fiber terminals in *GPHR^F/F^;GluD2-Cre* mice. Triple immunofluorescence for mGluR1α (green), vGlut1 (red), and calbindin (Calb; cyan) in cerebellar sections of *GPHR^F/+^;GluD2-Cre* mice and *GPHR^F/F^;GluD2-Cre* mice at P60. Asterisks indicate PC somata. Scale bar: 20 µm. For ***A****−****C***, three mice per genotype were used; *n* = 5 sections per mouse; representative images are shown.

Reduced GABAergic transmission from BCs impairs elimination of CF synapses, which leads to abnormal innervation of CF synapses onto PCs (Nakayama et al., 2012). Our morphological and electrophysiological analyses confirmed the disruption of inhibitory inputs from BCs. Thus, we hypothesized that CF innervation was altered in *GPHR^F/F^;GluD2-Cre* mice. To test this hypothesis, immunostaining for calbindin and vGlut2, a marker for CF axon terminals, was performed. In normal mice, vGlut2-positive CF terminals were distributed in the molecular layer and associated with PC dendrites after elimination (Miyazaki et al., 2003). In both *GPHR^F/F^;L7-Cre* and control mice, vGlut2-positive CF terminals exclusively innervated spino-dendritic domains in the molecular layer. However, in *GPHR^F/F^;GluD2-Cre* mice, CF terminals were frequently associated with PCs soma in addition to dendrites (Fig. 8*A*, arrowheads). Quantification of the vGlut2 puncta on the PC somata revealed a significant increase in *GPHR^F/F^;GluD2-Cre* mice when compared with that of control and *GPHR^F/F^;L7-Cre* mice (Table 1; control: 9.95 ± 1.07%; *GPHR^F/F^;GluD2-Cre*: 69.02 ± 4.21%; *GPHR^F/F^;L7-Cre*: 15.06 ± 1.30%; three mice per group; five sections per mouse; *n* = 15 sections for each group; *GPHR^F/+^;GluD2-Cre*: 172 PCs; *GPHR^F/F^; GluD2-Cre*: 141 PCs; *GPHR^F/F^; L7-Cre*: 162 PCs; one-way ANOVA, *F*_(2,42)_ = 156.4, *p* < 0.0001; and Sidak’s *post hoc* test, control vs *GPHR^F/F^;GluD2-Cre*, **p < 0.0001, control vs *GPHR^F/F^;L7-Cre*, *p* = 0.4383, *GPHR^F/F^;GluD2-Cre* vs *GPHR^F/F^;L7-Cre*, **p < 0.0001).

Next, to investigate whether somatic spines contacting with CF terminals occur on the surface of PCs in *GPHR^F/F^;GluD2-Cre* mice, we performed triple immunofluorescence staining for metabotropic glutamate receptor 1α (mGluR1α), vGlut2 and calbindin. At the CF-PC synapses, vGlut2 and mGluR1α are markers for presynaptic and postsynaptic proteins, respectively. As hypothesized, mGluR1α-positive punctae overlapped with vGlut2-positive punctae in perisomatic regions of PCs from *GPHR^F/F^;GluD2-Cre* mice, indicating CF innervation to somatic spines (Fig. 8*B*). Quantification confirmed that mGluR1 and vGlut2 double-positive puncta on the PC somata were increased significantly in *GPHR^F/F^;GluD2-Cre* mice when compared with that of control mice (Table 1; *GPHR^F/+^;GluD2-Cre* at P60: 9.34 ± 1.27%; *GPHR^F/F^;GluD2-Cre* at P60: 45.73 ± 4.016%; three mice per group; five sections per mouse; *n* = 15 sections for each group; *GPHR^F/+^;GluD2-Cre* at P60: 172 PCs; *GPHR^F/F^;GluD2-Cre* at P60: 141 PCs; unpaired Student’s *t* test, *t*_(28)_ = 8.648, *p < 0.0001). In contrast, there was no apparent change in the distribution of vGlut1-positive parallel fiber terminals (Fig. 8*C*).

**Figure 9.**
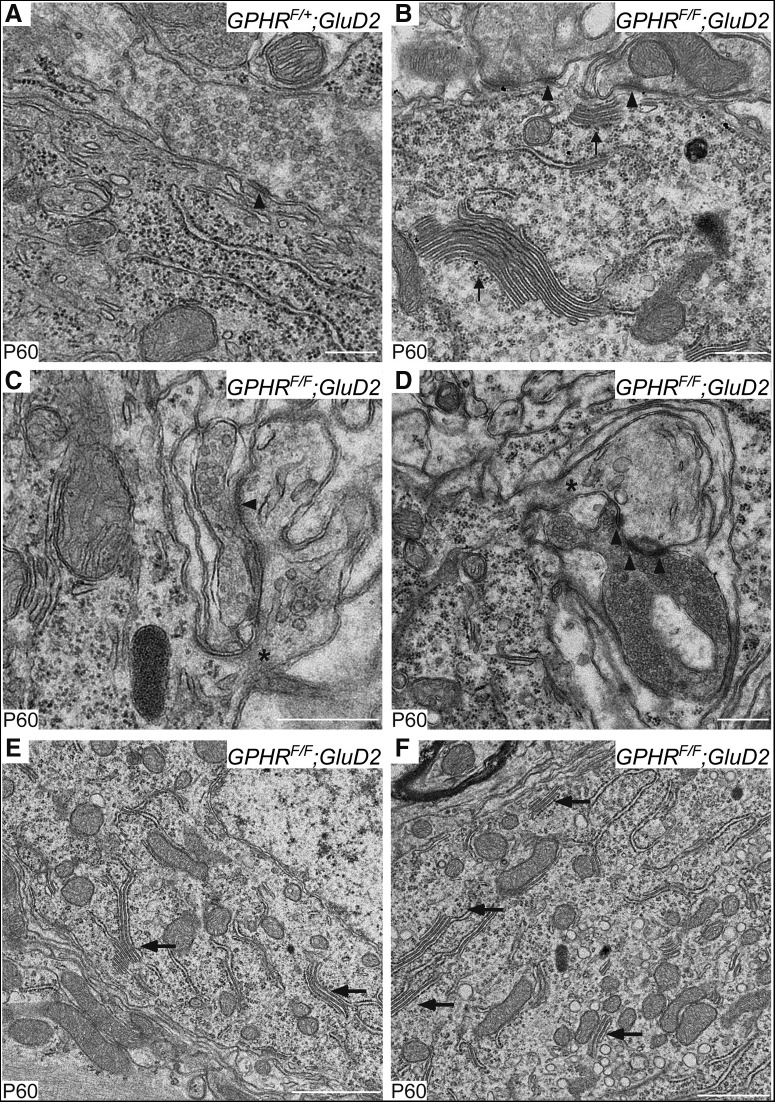
CF innerved to PC soma in *GPHR^F/F^;GluD2-Cre* mice. Electron micrographs of PC soma in *GPHR^F/+^;GluD2-Cre* (***A***) and *GPHR^F/F^;GluD2-Cre* mice (***B****−****F***) at P60. Arrowheads indicate active zones. Arrows indicate dense stacks of the cisternae of the smooth endoplasmic reticulum. Asterisks indicate necks of the spine from PC soma. Scale bars: 500 nm. ***A****−****F***, Three mice per genotype were used; *n* = 5 sections per mouse; representative images are shown.

Using EM analysis, synaptic contacts on PC somata were observed directly. Some typical BC innervations on PC soma were identified in control and *GPHR^F/F^;GluD2-Cre* mice (Fig. 9*A*,*B*). Consistent with immunofluorescence analysis (Fig. 8*B*), we further observed CF terminals forming synapses with somatic spines of PCs in *GPHR^F/F^;GluD2-Cre* mice (Fig. 9*C*,*D*). Moreover, we frequently observed lamellar bodies, dense stacks of smooth ER, in the perikarya of PCs from *GPHR^F/F^;GluD2-Cre* mice (Fig. 9*B*,*E*,*F*, arrows). These structures contain inositol 1,4,5-triphosphate receptors and become abundant within dendrites in response to stimulation through mGluRs (Yamamoto et al., 1991; Banno and Kohno, 1996). The number of lamellar bodies per nm^2^ of the perikarya of PCs increased significantly in *GPHR^F/F^;GluD2-Cre* mice when compared with that of *GPHR^F/+^;GluD2-Cre* mice (Table 1; control: 0.29 ± 0.06, *n* = 5; *GPHR^F/F^;GluD2-Cre*: 6.49 ± 0.55, *n* = 5; unpaired Student’s *t* test, *t*_(8)_ = 11.27, *p* < 0.0001). Thus, these data suggest altered intracellular structures because of CF innervation on PCs soma in *GPHR^F/F^;GluD2-Cre* mice.

## Discussion

In this study, the crucial role of luminal acidification of the Golgi apparatus in maintaining normal structural integrity of the Golgi apparatus in neurons is reported. Although the relationship between Golgi pathology and neurodegeneration has been suggested based on the genetic analysis of Golgi-related proteins, no studies have thoroughly analyzed abnormalities of cerebellar neuronal circuits on perturbations in luminal pH of the Golgi apparatus *in vivo* (Alvarez-Miranda et al., 2015). Since available pharmacological blockers lack specificity for manipulation of the luminal pH of the Golgi apparatus, we believe that genetically modified mouse models are currently an ideal tool to study the relationship between luminal pH and morphology of the Golgi apparatus.

We demonstrated previously that the luminal pH of the Golgi apparatus is altered in *GPHR* mutant Chinese hamster ovary cells (Maeda et al., 2008). In the present study, using a pH-sensitive GFP variant, pHluorin-Golgi, we revealed that the luminal pH of the Golgi apparatus is elevated in primary cultured neurons that no longer express normal levels of GPHR. Furthermore, it has been reported that the pH gradient between the ER and the Golgi apparatus disappears by RNA silencing of GPHR in HeLa cells (Vavassori et al., 2013). Taken together, these results support the concept that GPHR is both necessary and sufficient for the maintenance of the Golgi luminal acidic pH and no other mechanism can compensate for pH gradient maintenance in neurons.

Our results revealed that loss of *GPHR* markedly increased the degeneration of PCs. This, in turn, leads to the reduction of cerebellar size and might contribute to the abnormal motor phenotype observed in *GPHR^F/F^;GluD2-Cre* mice. Interestingly, the loss of PCs stands out in the lobule IX. However, this observation is in sharp contrast with other studies that reported a patterned or area selective PC degeneration (Sarna and Hawkes, 2003; Cerminara et al., 2015). In addition, it has also been reported that lobules IX and X exhibit some level of neuroprotection (Sarna and Hawkes, 2003, 2011). In future, it would be interesting to analyze the factors that confer lobule-dependent neuroprotection in different mouse models.

The observation that PCs begin to degenerate at P60 in *GPHR^F/F^;GluD2-Cre* mice demonstrates that the luminal acidic pH of the Golgi apparatus under physiological conditions is essential for PC survival. Impaired luminal acidification of the Golgi apparatus due to deletion of GPHR causes structural disorganization of the Golgi apparatus and results in neurodegeneration. Notably, PCs from *GPHR^F/F^;GluD2-Cre* mice still exhibit normal organization of the Golgi apparatus with GPHR immunoreactivity until at least P30, indicating that maturation in the early developmental stage of the cerebellum occurs normally in these PCs. In other words, the abolishment of GPHR expression was completed only after stabilization of cerebellar circuits. The motor phenotypes because of PC loss in *GPHR^F/F^;GluD2-Cre* mice were comparable with other mouse models where Golgi-associated proteins such as *Rer1* and *GM130* are ablated (Liu et al., 2017; Valkova et al., 2017). Loss of GM130 starts from early developmental stages and results in atrophic dendrites and eventual disruption of PCs. This indicates that the proper function of the Golgi apparatus contributes to PC maturation (Liu et al., 2017). However, our current study also suggests that luminal acidification of the Golgi apparatus is also responsible for PC survival after maturation.

Our previous *in vitro* study showed that GPHR plays an important role in maintaining the morphological integrity of the Golgi apparatus (Maeda et al., 2008). In addition, morphological abnormalities in the Golgi apparatus have also been reported in fibroblasts derived from patients with mutations in the gene encoding ATPV0A2, which is involved in luminal acidification of the Golgi apparatus (Hucthagowder et al., 2009; Foulquier et al., 2012). Consistent with these studies, our morphological analyses show structural abnormalities of the Golgi apparatus in GPHR-deficient PCs. Interestingly, vesiculation and fragmentation of the Golgi apparatus are pathological hallmarks of various neurodegenerative diseases including amyotrophic lateral sclerosis, Parkinson’s, Alzheimer’s, and Huntington’s diseases (Fan et al., 2008; Haase and Rabouille, 2015; Ayala and Colanzi, 2017). In addition, various models for human neurodegenerative diseases imply that fragmentation of the Golgi apparatus in neurons occurs as a result of the dysregulation of ER-Golgi transport (Huynh et al., 2003; Joshi et al., 2014). Together, our findings suggest that GPHR plays important roles, including morphological maintenance of the Golgi apparatus in neurons. However, genetic screening has not identified GPHR as a causative gene for human diseases. Nonetheless, our present study suggests a causative link between fragmentation of the Golgi apparatus and neurodegeneration. We cannot exclude the possibility that an abnormality of the ER secondarily leads to the abnormal morphology of the Golgi apparatus due to GPHR deficiency. Further studies will be required to elucidate whether transport between ER and the Golgi apparatus is also impaired in GPHR-deficient neurons.

Loss of GM130 and Rer1 proteins in premature PCs affects dendrite and axon morphology, respectively, indicating that the proper function of the Golgi apparatus is important for intact PC polarization (Liu et al., 2017; Valkova et al., 2017). In our study, there was apparently no change in the morphology of PC dendrites in *GPHR^F/F^;GluD2-Cre* mice, whereas axonal swelling in GPHR-deficient PCs was observed at P45, and the number and size of axon swellings increased with age. Our observation is in accordance with those from previous studies in mouse models where PCs lack Golgi-associated proteins (Watanabe et al., 2010; Valkova et al., 2017). However, it is unlikely that the axonal swellings in GPHR-deficient PCs are related to myelin abnormality as observed in PCs deficient in ceramide glucosyltransferase, a Golgi-associated enzyme (Watanabe et al., 2010), because myelination of GPHR-deficient PC axons appears normal. Furthermore, we also noticed that a larger portion of the soma of DCN neurons of *GPHR^F/F^;GluD2-Cre* mice were surrounded with intercellular spaces because of the loss of axosomatic synapses as early as P45. Importantly, the synaptic terminals of BCs in *GPHR^F/F^;GluD2-Cre* mice were also lost at P60, which strengthens our notion that impairment of luminal acidification of the Golgi apparatus is closely associated with axonal and synaptic pathology. Axonal swelling is a hallmark of axonal dystrophy, which can lead to neurodegeneration (Coleman, 2005). Major causes for axon degeneration converge in the dysfunctions of axonal transport, the autophagy-lysosome system, mitochondria, and increased intra-axonal calcium (Coleman, 2005; Komatsu et al., 2007; Nishiyama et al., 2007; Liang et al., 2010; Koike et al., 2017; Yamaguchi et al., 2017). Although the direct link between impairment of luminal acidification of the Golgi apparatus and axon and synaptic abnormalities remains unclear, it is possible that loss of *GPHR* is associated with axonal transport dysfunction.

Intriguingly, CF innervation on PC soma has been observed in PC-specific deletion of *GluD2* and *RORa* because of territorial changes in parallel fibers and CFs (Miyazaki et al., 2010; Chen et al., 2013). Furthermore, reduced GABAergic transmission from BCs leads to abnormal elimination of CF synapses onto PCs during early developmental stages (Nakayama et al., 2012). However, the role of GABAergic transmission in maintaining proper CF innervation in the adult cerebellum has not been examined. In our mouse model, CF innervates the PC soma instead of the BC axonal terminal territory after stabilization of the cerebellar circuits. This novel finding suggests that, in addition to heterosynaptic competition of excitatory innervations, the inhibitory-excitatory imbalance can also result in a redistribution of the innervation territory on mature PCs. Further studies are required to understand the roles of BCs in maintenance of proper CF innervation onto PCs by use of BC-specific *Cre* mouse lines.

What could be the pathological consequences of altered cerebellar neuronal circuitry in *GPHR^F/F^;GluD2-Cre* mice? Our morphological data showed that the formation of somatic spines in GPHR-deficient PCs and the targeting of CFs to these somatic spines as well as the electrophysiological data suggesting disrupted and nonfunctional inhibitory BCs hint at a possible excitatory-inhibitory imbalance in these PCs. Unlike the normal situation, where CF inputs are created only in dendrites, the direct excitation of the soma by CF implies that voltage changes are minimal and thus result in a dramatic increase in the PC membrane potential. Moreover, the severity of depolarization around the PC soma is maintained because inhibition from BC terminals no longer exists. As calcium permeable channels open because of depolarized membrane potentials, overexcitation may create an unnecessarily large calcium load in the PC cytoplasm, leading to calcium excitotoxicity. This may lead to the eventual disruption and death of PCs. Indeed, PCs from *GPHR^F/F^;GluD2-Cre* mice possess dense stacks of smooth ER, which prevent excessive Ca^2+^ release from the reservoir (Banno and Kohno, 1996) and respond to calcium excitotoxicity because of the excitatory-inhibitory imbalance. Nevertheless, GPHR-deficient PCs are largely lost with age, suggesting that neurodegeneration is likely caused by impairment of luminal acidification of the Golgi apparatus. Further studies must be conducted to elucidate the function of the Golgi apparatus for maintaining the integrity of neuronal circuits.

## Synthesis

Reviewing Editor: Pablo Castillo, Albert Einstein College of Medicine Decisions are customarily a result of the Reviewing Editor and the peer reviewers coming together and discussing their recommendations until a consensus is reached. When revisions are invited, a fact-based synthesis statement explaining their decision and outlining what is needed to prepare a revision will be listed below. The following reviewer(s) agreed to reveal their identity: Roy Sillitoe, Alanna Watt. Note: If this manuscript was transferred from JNeurosci and a decision was made to accept the manuscript without peer review, a brief statement to this effect will instead be what is listed below.

Your manuscript was evaluated by two experts in the field. They agree your study makes a significant contribution on the role of the Golgi apparatus in the cerebellum with a potential relevance to disease. Both reviewers understand the results are convincing and elegant, and that the revised manuscript properly addressed prior criticisms. However, as you will see below, they also raised a number of comments regarding the text --i.e. grammar and logic of several of your statements. Before this manuscript can be considered for publication, you must revise the text thoroughly. Please mark all new changes in red.

REVIEWER #1

The authors have provided a revised version of their manuscript. Substantial effort was taken to address the previous reviewers comments, and in particular the authors have added a number of statistical measures based on the recommendations of the reviewers. The paper appears considerably stronger.

Overall, I think this is a very interesting manuscript and I share the enthusiasm of the previous reviewers in that the scientific problem that is addressed is important. The techniques are appropriate and appear to be carried out with care. However, I have some suggestions that should be addressed:

1) The major concern that I have is that the grammar MUST be fixed throughout the paper. I will only give some examples below - I will leave it up to the authors to address this carefully and go through several rounds of editing of their paper. This is an issue not only for the readability of the paper, but in many cases it is the grammar itself that causes the scientific statements and conclusions to become obscured.

2) Use Golgi apparatus throughput the paper rather than just “Golgi”.

3) In the Abstract, you state “...functional abnormalities of neuronal circuit”. Circuits should be plural.

4) Also in the Abstract, “In addition, innervation pattern...” should be “the innervation”

5) In the Abstract, the sentence should read “mutant PCs in the mature cerebellum”

6) The authors state “Intriguingly, after proper maturation of cerebellar circuits,...”. What is the evidence for this? How did you directly test for maturation of the cerebellar circuits? This is important to establish because your claim is that cells die after this.

7) The authors state “...for maintenance of proper neuronal morphology”. What exactly are your referring to by morphology? Sub-cellular or cellular level? You need to be specific. Sounds like there is a critical impact on sub-cellular morphology, but your data may also suggest that dendrites etc (neuronal morphology) eventually are compromised. Please clarify with more explicit statements.

8) The authors state “...function of PCs and BCs, whereas altered cerebellar circuits may facilitate the loss of PCs.” I am not sure what you mean by this.

9) What is your justification for using heterozygous mutant mice as controls? Are you not afraid of haploinsufficiency effects?

10) I have no idea what you mean by “Axons shoes area...”

11) What is the justification for using only female mice for the rotarod experiments? Were males and females used in other experiments?

12) The numbers of mice used for the clasping, open field etc was especially low. Only 3 mice per genotype. I realize there were issues with breeding, but I needed to raise this issue. I suppose its fine since the effect was reliable.

13) I am not sure what you mean by “Elevateion of Golgi”

14) For the virus experiments, how do you test for equal infection across the genotypes and across experiments? What is your measure of consistency?

15) In more than one case you state “...mice showed motor coordination disorder...” It is better to refer to these as mouse phenotypes (and not disorders) since you did not actually measure the defects in regards to neurological deficits per se.

16) Why was the microglia analysis done in lobule IX? This is puzzling since lobules IX and X typically exhibit neuroprotection in many models of ataxia and other disorders - see Sarna and Hawkes, 2003

17) The authors state “..for calbindin but negative for vesicular GABA transporter (vGAT)..” Okay, so what is the identity of these terminals/cells?

18) It is not so clear what cell types in the cerebellum express Neuroligin2

19) The authors state “...satellite cell axon terminals...” but I think you mean stellate cell

20) The authors state “...infiltration of electron-lucent Bergmann glia (BG) processes was observed instead...” I don't quite follow what you think has happened here

21) “To prove this hypothesis” should be changes to “To test....”

22) The authors state “type-2 vesicular glutamate transporter (vGlut2), a specific marker for CF axon terminals.” This is not accurate since it also labels mossy fiber terminals.

23) the authors state “CF terminals were occasionally....” Based on your numbers, its is more than occasional!

24) Please define what “spine protrusions” are.

25) The authors state “staining for metabotropic glutamate receptor 1α (mGluR1α), vGlut2 and calbindin...”, but they have not explained what they were looking for in this relationship

26) In the Discussion, I do not understand what you mean by “two GPHR genes in the human genome and thus the unlikeliness of loss of function.”

27) In the Discussion, the state “It is necessary to high light in tow major differences in mammalian and Drosophila GPHR.” First, please fix the grammar. Second, why is it necessary to compare the species? I do not follow the logic of the argument. This section needs to be integrated into the rest of the discussion in a better way.

28) The authors state “indicating that neurodegeneration is caused only by impairment of Golgi luminal acidification..” This is an overly strong statement, and I suspect this was unintentional. I think the grammar just needs to be fixed.

REVIEWER #2

This is an interesting study that looks at the effect of changes in Golgi pH on cerebellar circuit and cellular properties, which has relevance for several disease models. The authors use a combination of immunocytochemistry, quantitative EM, electrophysiology and behaviour to address this question in great depth. The study is convincing and elegant. This is a careful and interesting study that has been significantly improved by the reviews and the new data and text that has been added in response to them.

I have just a few comments, mostly stylistic, although the first comment is not really minor and should be confirmed.

Major comments

1. In figure 2D, are the traces coloured incorrectly? From my reading of the Methods and the text, it is the GPHR_F/F mice that have ataxia, which is reflected in higher scores (which would be consistent with lower scores for rotarod). But the score goes up for the control mouse. Please check. If the data is correct, it should be discussed in the text in more detail.

Minor comments:

1. Use of the word “Unequivocal” should be avoided in science, in my opinion. Biology is complex and few things can be said to be shown unequivocally. This is used in the Abstract (line 13) but also elsewhere (line 588). Please change.

2. Line 214: “the ‘n’ refers to the density of Purkinje cells”. I don't think this is true -- you state in the text what n is (it is often the number of sections for immunocytochemistry). Please clarify, perhaps just saying n is stated in the text for each comparison.

3. On a related note, sometime you state “n = X section” while other times you say “n = X sections”. I think that plural is clearer. Either way, it should be standardized throughout the text.

3. Line 622 “high light in tow” should be “highlight two”

4. The relevant text in the figures that allows discrimination of control and mutant mice is *very* small in many places since it is in superscript. This could be illegible in print, and should be sized up for clarity (or if there is some way to show this that is *not* superscript, it would certainly help with the problem).
